# Exosome‐Based Theranostic for Gastrointestinal Cancer: Advances in Biomarker Discovery and Therapeutic Engineering

**DOI:** 10.1002/smtd.202402058

**Published:** 2025-02-25

**Authors:** Mohit J. Mehta, Dongjun Shin, Hyun Sung Park, Jun Su An, Seong Il Lim, Hyun Jin Kim, Seungpyo Hong, Jiyoon Bu

**Affiliations:** ^1^ Department of Biological Sciences and Bioengineering Inha University 100 Inha‐ro, Michuhol‐gu Incheon 22212 Republic of Korea; ^2^ Adult Stem Cell Group Faculty of Medicine and Health Technology Tampere University Tampere Finland; ^3^ Department of Biological Engineering Inha University 100 Inha‐ro, Michuhol‐gu Incheon 22212 Republic of Korea; ^4^ Biohybrid Systems Research Center Inha University 100 Inha‐ro, Michuhol‐gu Incheon 22212 Republic of Korea; ^5^ Pharmaceutical Sciences Division and Wisconsin Center for NanoBioSystems (WisCNano) School of Pharmacy University of Wisconsin – Madison 777 Highland Ave Madison WI 53705 USA; ^6^ Yonsei Frontier Lab and Department of Pharmacy Yonsei University 50 Yonsei‐ro Seodaemun‐gu Seoul 03722 Republic of Korea

**Keywords:** exosomes, extracellular vesicles, exosome engineering, exosomal biomarkers, gastrointestinal cancer

## Abstract

Exosomes have emerged as versatile biomolecules in gastrointestinal (GI) cancer management, leveraging their inherent cargo‐carrying capabilities to overcome the limitations of current diagnostic and therapeutic strategies. This review provides a comprehensive exploration of exosome‐based technologies, highlighting their dual roles as diagnostic biomarkers and therapeutic vehicles. On the diagnostic front, this review investigates specific exosomal biomarkers—including miRNAs, lncRNAs, circRNAs, and proteins—emphasizing their roles in tumor biology and their clinical utility, such as diagnostic accuracy, early detection potential, and prognostic significance. Therapeutically, this study examines the sources, purification methodologies, and engineering strategies that enhance the therapeutic efficacy of exosomes in modulating immune responses, overcoming drug resistance, and suppressing metastasis. By providing a comprehensive overview of the field, this review serves as a guideline for researchers and clinicians entering this field, offering novel insights into the potential of exosome‐based theranostic systems and delineating future directions to overcome existing challenges.

## Graphical abstract

This review explores the dual roles of exosomes in gastrointestinal (GI) cancers as diagnostic biomarkers and therapeutic agents. It highlights advancements in exosomal miRNAs, lncRNAs, circRNAs, and proteins for cancer detection and prognosis. The study also examines engineering strategies for exosome‐based drug delivery systems, their potential to inhibit growth, reverse drug resistance, modulate immunity, and addresses challenges in clinical translation.

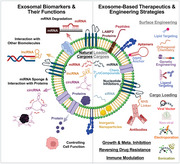

## 1 Introduction

Gastrointestinal (GI) cancers, which include anal, colorectal, liver, pancreatic, gastric, and small intestine cancer, contribute to 25.8% of global cancer incidence and account for 35.4% of cancer‐related deaths.^[^ ^1^ ^]^ Despite advances in diagnostic and therapeutic techniques, the early stages of the disease often present without clinical symptoms, causing GI cancers to remain undetected until later stages. Consequently, diagnoses frequently occur at an advanced stage, leading to a relatively low five‐year survival rate due to the limited availability of effective treatment options at this stage.^[^ ^2^ ^]^ Specifically, the failure of definitive treatment and patient mortality predominantly arise from metastasis and recurrence.^[^ ^2^ ^]^ Although existing biomarkers and drug delivery systems have shown potential, their effectiveness remains limited, particularly in detecting early stage disease and treating advanced‐stage cancer. Therefore, it is essential to identify new biomarkers for early diagnosis and prognosis monitoring while developing effective treatment strategies to prevent recurrence and control metastasis.

Among various biomolecules utilized for diagnosing and treating GI cancer, extracellular vesicles (EVs) have gained substantial scientific and clinical attention due to their role in cell‐to‐cell communication. EVs are lipid bilayer‐enclosed nanoparticles released by most cell types.^[^ ^3^ ^]^ These vesicles facilitate intercellular communication, allowing cells to exchange molecular information and influence their microenvironment. Specifically, their ability to transport bioactive molecules, including proteins, nucleic acids, and lipids, makes them promising candidates for biomarker discovery and targeted therapy in oncology.

Cells release different types of EVs, which include apoptotic bodies, microvesicles (also known as ectosomes), and exosomes.^[^ ^4^ ^]^ These vesicles are classified based on their biogenesis pathway, size, flotation density on a sucrose gradient, lipid composition, sedimentation force, and protein contents. Among these subtypes, exosomes have attracted significant scientific interest due to their potential as natural nanocarriers or biomarkers for diagnosing various diseases. Exosomes exhibit unique biogenetic pathways that enable them to selectively incorporate molecular cargo from their parental cells. Specifically, unlike other EVs that originate from the shedding of the plasma membrane, exosomes are derived from an inward budding of a specific type of endosomes known as multivesicular bodies (MVBs), followed by fusion with the plasma membrane.^[^ ^5^ ^]^ Consequently, exosomes carry various bioactive molecules from their cell of origin, including transmembrane and cytosolic proteins and small nucleic acids (mRNA, miRNA, and DNA fragments). These cargoes are delivered to recipient cells via direct fusion and internalization through endocytosis or receptor‐ligand binding.

The transportation of exosomal proteins and nucleic acids to recipient cells may modulate various physiological and pathological processes. Specifically, regarding exosomal proteins, these cargoes are known to regulate innate immunity, cell adhesion, cell structure, membrane fusion, metabolism, and signal transduction.^[^ ^6^ ^]^ For instance, exosomes derived from activated antigen‐presenting cells (APCs) contain substantial amounts of major histocompatibility complex (MHC) class I and II molecules, which enhance antigen presentation in recipient APCs.^[^ ^7^ ^]^ Additionally, MHC‐I or MHC‐II on the exosome surface can directly activate natural killer (NK) cells through interactions with the NKG2D ligand, suggesting their role in immune modulation and potential immunotherapeutic applications for GI cancer.^[^ ^8^ ^]^

Similarly, exosomal nucleic acids significantly influence recipient cells and their microenvironment. For example, exosomal microRNAs (miRNAs) and their inhibitors regulate gene expression in recipient cells, affecting multiple cellular pathways.^[^ ^8^ ^]^ In the context of cancer, tumor‐derived exosomes carrying specific nucleic acids modulate the phenotype of surrounding cells by altering gene expression networks that drive tumor progression and metastasis.^[^ ^8^ ^]^ The delivery of these small nucleic acid fragments via exosomes plays a crucial role in genetic communication between cells, influencing the genomic landscape of the recipient cells. For instance, exosomes from gastric cancer (GC) cells have been shown to activate the PI3K/Akt and MAPK/ERK pathways, promoting tumor cell proliferation.^[^ ^9^ ^]^ Understanding these mechanisms offers valuable insights into the role of exosomal nucleic acids in cancer progression, highlighting their significance in intercellular communication.

**Figure 1:**
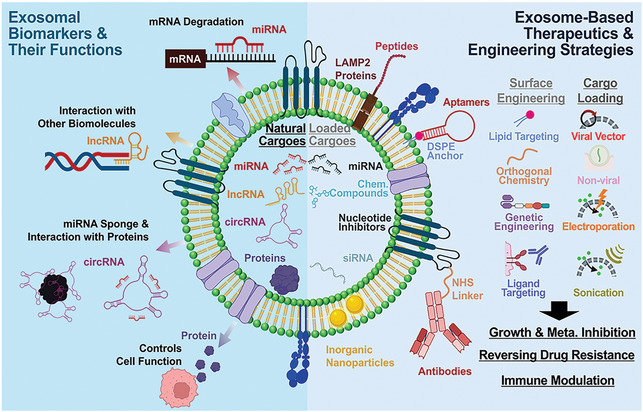
Exosome‐based theranostics for gastrointestinal (GI) cancer: exosomal biomarkers for the diagnosis of GI cancer and engineered‐exosomes for enhanced GI cancer therapeutics.

Owing to their distinctive functionalities and characteristics, numerous studies have explored the use of specific exosome subtypes as diagnostic biomarkers and drug delivery carriers. Specifically, advances in exosome purification and intravesicular molecular analysis have revealed their substantial potential for clinical applications. This review focuses on EVs, specifically exosomes, recent advancements in EV‐based technologies, and their application in diagnostic and therapeutic contexts within GI cancer (**Figure** **1**). This review will highlight the key features that make exosomes valuable in oncology, emphasizing their role in intercellular communication. We will examine studies that demonstrate the potential of exosomes as biomarkers for GI cancer diagnosis and prognosis, particularly focusing on intravesicular constituents such as proteins, miRNAs, lncRNAs, and circRNAs. Following their role as diagnostic biomarkers, we will explore the potential of exosomes as therapeutic agents, particularly in inhibiting metastasis, overcoming drug resistance, and modulating immune responses. Lastly, this review will provide insights into the rapidly evolving clinical applications of exosome‐based strategies, addressing existing challenges in clinical translation and suggesting multidisciplinary approaches to overcome these limitations. By critically evaluating current advancements, challenges, and potential strategies to overcome limitations, this review aims to provide a comprehensive perspective on exosome‐based strategies for GI cancer management, serving as a guideline for researchers and clinicians new to the field.

## 2 Exosomes as a Biomarker for Gastrointestinal Cancers

### 2.1 Exosomal miRNAs

#### 2.1.1 Exosomal miRNAs as a Diagnostic Biomarker for GI Cancer

Exosomes contain miRNAs, lncRNAs, circRNAs, and proteins derived from their parental cells, which can be utilized for the diagnosis and prognosis of various diseases. Notably, exosomal miRNAs are among the most frequently studied biomarkers for GI cancer diagnosis. These miRNAs play a crucial role in post‐transcriptional gene regulation by binding to target mRNAs, leading to their degradation or translation inhibition. Encapsulated within the protective lipid bilayer of exosomes, miRNAs are shielded from enzymatic degradation, making them stable in various biological fluids, including blood, urine, and saliva. This stability, along with their ability to reflect the molecular state of the originating cells, has garnered significant interest in using exosomal miRNAs as non‐invasive biomarkers for disease detection, particularly in cancers. Specifically in GI cancers, exosomal miRNAs have been shown to participate in tumor progression, metastasis, and drug resistance, highlighting their potential utility for early diagnosis, prognosis, and monitoring therapeutic responses. Accumulating evidence has demonstrated the high specificity and sensitivity of exosomal miRNAs in detecting cancer‐related alterations across various types of GI cancer.^[^ ^10^ ^]^

#### 2.1.2 Clinical Utility of Exosomal miRNA for the Diagnosis of GI Cancer

Numerous studies have highlighted the importance of exosomal miRNAs as a biomarker for GI cancer (**Table** **1**). In a study conducted by Liu et al., the group evaluated the prognostic value of 145 serum exosomal miRNAs. The authors observed that the expression level of exosomal miR‐4772‐3p exhibited a strong correlation with tumor recurrence after adjuvant therapy in patients with stage II and III colon cancer. Lower levels of miR‐4772‐3p were associated with an increased risk of recurrence, exhibiting an AUC‐ROC of 0.72 (p = 0.001) for predicting tumor recurrence.^[^ ^11^ ^]^ In another study, Teng et al. investigated the impact of exosomes on both donor and recipient cells by examining exosomal miRNA profiles in primary mouse colon tumors and liver metastasis of colon cancer. Their findings revealed that the tumor suppressor miRNA miR‐193a, when prevented from entering exosomes due to the knockout of major vault protein (MVP), accumulated in donor cells and inhibited tumor progression. Elevated levels of circulating exosomal miR‐193a were thus associated with advanced disease, highlighting the role of MVP in selectively sorting miRNAs and promoting tumor progression.^[^ ^12^ ^]^

**Table 1:** Summary of the clinical studies that utilized exosomal miRNAs as a biomarker for gastrointestinal (GI) cancer.

Patients	Specimen	Exosome isolation	RNA xtraction	miRNAs	Profiling	Clinical outcomes	Refs.
84 CC (colon cancer)	Serum	ExoQuick	SeraMir Exosome RNA Purification/ Amplification Kit	145 miRNAs (miR‐4772‐3p)	RNA‐seq, quantitative reverse transcription polymerase chain reaction (PCR)	Reduced expression of exosomal miR‐4772‐3p predicts recurrence and poor response to FOLFOX adjuvant therapy	[11]
40 CC	Plasma	exoEasy Maxi Kit	exoRNeasy Serum/Plasma Midi kit	miR‐193a, miR‐126, miR‐148a, miR‐196b	miRNA microarray, PCR	Higher level of exosomal miR‐193a is associated with an increased risk of liver metastasis	[12]
88 CRC (colorectal cancer) 11 HD	Serum	Ultracentrifugation (UC)	RNeasy Mini Spin Columns	164 miRNAs (let‐7a, miR‐1229, miR‐1246, miR‐150, miR‐21, miR‐223, and miR‐23a)	miRNA microarray, PCR	Higher levels of exosomal let‐7a, miR‐1229, miR‐1246, miR‐150, miR‐21, miR‐223, and miR‐23a found in CRC patients	[13]
25 CRC 13 HD	Serum	ExoQuick	SeraMir Exosome RNA Amplification kit	11 miRNAs	PCR	Higher levels of exosomal miR‐23a and miR‐301a found in CRC patients	[14]
168 CRC 20 HD	Serum	Total Exosome Isolation Kit	miRNeasy mini kit	miR‐6803‐5p	miRNA microarray, PCR	Higher levels of exosomal miR‐6803‐5p are associated with advanced disease stage, metastasis, and poorer survival outcomes	[15]
133 CRC 60 HD	Serum	ExoQuick	miRNeast Serum Kit	miR‐150‐5p	PCR	Reduced expression of exosomal miR‐150‐5p correlates with poor differentiation and metastasis	[16]
54 HCV (hepatitis C virus) 44 HBV (hepatitis B virus) 19 HD	Plasma	ExoEnrich instant exosome isolation kit	TRIzol	miR‐10b‐5p/miR‐221‐3p/miR‐223‐3p/miR‐21‐5p	RNA‐seq/qRT‐PCR analysis	Higher levels of exosomal miR‐21‐5p, miR‐10b‐5p/miR‐221‐3p/miR‐223‐3p found in hepatocellular carcinoma (HCC) patients	[17]
55 PDAC (pancreatic ductal adenocarcinoma)	Plasma	UC	miRNeasy serum/plasma kit/ miRNeasy Mini Kit	miR‐4525, miR‐451a and miR‐21	miRNA microarray/qRT‐PCR	High expression of miR‐4525, miR‐451a, and miR‐21 in portal vein blood (PVB) is associated with recurrence and poor survival	[18]
135 GC 23 HD	Plasma	UC	miRNeasy serum/plasma kit/miRNeasy Mini kit	miRNA‐21, miRNA‐92a,	miRNA array	High levels of ex‐miR‐21 and low levels of ex‐miR92a in stage II and III patients are associated with worse overall survival (OS) and peritoneal recurrence‑free survival (PRFS)	[19]

Other miRNAs are also being investigated as biomarkers for GI cancer. Ogata‐Kawata et al. identified seven exosomal miRNAs (let‐7a, miR‐1229, miR‐1246, miR‐150, miR‐21, miR‐223, and miR‐23a) that were significantly elevated in colorectal cancer (CRC) patients. Interestingly, the expression levels of these miRNAs were reduced upon tumor resection. These miRNAs also demonstrated higher secretion levels in colon cancer cell lines, confirming the potential of these exosomal miRNAs to be utilized as a biomarker for CRC.^[^ ^13^ ^]^ Similarly, exosomal miR‐23a and miR‐301a were found to be significantly elevated in CRC patients. Although the expression levels of these miRNAs did not significantly correlate with the clinicopathological features of the patients, they differed between patients and healthy subjects, with AUC‐ROC values of 0.84 and 0.90 for exosomal miR‐23a and miR‐301a, respectively.^[^ ^14^ ^]^

In a study conducted by Yan and colleagues, exosomal miR‐6803‐5p levels were also significantly higher in CRC patients than in healthy controls, and were associated with later TNM stages, lymph node metastasis (LNM), and liver metastasis. Elevated levels of miR‐6803‐5p also correlated with poorer overall and disease‐free survival.^[^ ^15^ ^]^ In contrast, serum exosomal miR‐150‐5p levels were significantly reduced in CRC patients compared to healthy controls, and levels increased post‐surgery. Lower miR‐150‐5p levels correlated with poorer differentiation, LNM, and advanced TNM stage, while higher levels were associated with longer survival, indicating its potential as a prognostic biomarker for CRC.^[^ ^16^ ^]^

Exosomal miRNAs have also emerged as significant biomarkers for other GI cancers, including hepatocellular carcinoma (HCC), pancreatic cancer (PCa), and GC. For example, a study conducted by Ghosh et al. analyzed 41 deregulated miRNAs in hepatitis C virus (HCV) HCC and normal liver tissues. Their findings revealed that four miRNAs—miR‐21‐5p, miR‐10b‐5p, miR‐221‐3p, and miR‐223‐3p—were significantly upregulated in patient‐derived serum exosomes. ROC analysis indicated that the combined detection of these four miRNAs yielded an AUC‐ROC of 0.86 (chronic hepatitis vs. HCC) and 0.80 (non‐HCC vs. HCC), highlighting their strong potential as diagnostic biomarkers for HCC.^[^ ^17^ ^]^ In another study, Kawamura et al. evaluated exosomal miR‐4525, miR‐451a, and miR‐21 in portal vein and peripheral blood samples from 55 patients with pancreatic ductal adenocarcinoma (PDAC). Their findings indicated that these miRNAs were significantly upregulated in portal vein blood compared to peripheral blood. Consequently, elevated expression levels of exosomal miR‐4525, miR‐451a, and miR‐21 in portal vein blood were strongly associated with a high recurrence risk and poor overall survival (OS).^[^ ^18^ ^]^ For GC, Soeda et al. examined the expression levels of various exosomal miRNAs in patients with stage II and III GC. Their findings demonstrated that low expression of exosomal miR‐92a and high expression of exosomal miR‐21 were associated with worse OS and PFS.^[^ ^19^ ^]^

#### 2.1.3 Limitations and Prospects of Utilizing Exosomal miRNA as a Biomarker for GI Cancer

Exosomal miRNAs hold significant promise as biomarkers for GI cancers due to their stability, specificity, and ability to circulate in body fluids. These small RNAs are encapsulated within exosomes, which protect them from enzymatic degradation, thus providing a reliable medium for detecting cancer‐related miRNAs. Despite these advantages, challenges remain in their clinical application. A major limitation is in the lack of standardized, high‐throughput, and cost‐effective techniques for isolating and profiling exosomal miRNAs. Current isolation methods, such as ultracentrifugation (UC) and precipitation kits, often co‐isolate non‐exosomal components, reducing diagnostic accuracy.

Another challenge is the specificity of miRNAs. Although certain miRNAs are overexpressed in specific cancers, they can also be involved in other diseases, complicating the establishment of cancer‐specific signatures. Additionally, distinguishing exosomal miRNAs derived from cancer cells from those of normal cells is challenging, particularly in complex biological samples like blood, which further complicates their clinical application as diagnostic biomarkers

Addressing these limitations requires the development of more precise and scalable exosomal miRNA detection strategies. Recent advances in nanotechnology and biosensor development have shown potential in overcoming these challenges. For instance, nanomaterial‐based biosensors, isothermal amplification techniques, and microfluidic platforms have demonstrated high sensitivity and specificity in detecting exosomal miRNAs.^[^ ^20^ ^]^ Nanoparticle‐based detection, combined with electrochemical sensors, offers ultrasensitive detection capabilities by amplifying signals from even trace amounts of miRNAs. Additionally, integrating multistep purification processes into microfluidic devices may allow for faster and more automated isolation of exosomes from biological fluids.

Advancements in exosomal miRNA analysis technologies, such as NGS and machine learning algorithms, could further enhance both the sensitivity and specificity of miRNA‐based biomarkers. By utilizing these novel techniques, exosomal miRNA panels could be developed to differentiate between various GI cancers and other diseases, providing a more targeted approach to diagnosis and prognosis. Integration of exosomal miRNAs with other biomarker classes may also offer a more comprehensive understanding of the tumor microenvironment, enhancing their utility in clinical practice.

### 2.2 Exosomal lncRNAs

#### 2.2.1 Exosomal lncRNAs as a Diagnostic Biomarker for GI Cancer

LncRNAs are a class of non‐coding RNA transcripts longer than 200 nucleotides, produced through RNA polymerase II‐mediated transcription, but they lack the ability to encode proteins.^[^ ^21^ ^]^ These molecules are increasingly recognized as key regulators of gene expression and cellular processes. The mechanisms that govern the synthesis and maturation of lncRNAs, though still not yet fully understood, involve several essential processes. Notable among these are ribonuclease P‐induced cleavage, which produces mature ends, the assembly of small nucleolar RNA‐protein (snoRNP) complexes, and the formation of circular structures. These complex processes reflect the intricate regulation involved in lncRNA biogenesis. Current estimates suggest that the human genome encodes between 5400 and more than 10 000 lncRNA transcripts, a testament to their widespread presence and regulatory significance in various biological systems.^[^ ^22^ ^]^

The expression patterns of lncRNAs are highly specific to tissues and cells, with alterations in lncRNA levels often observed during the progression of numerous diseases, particularly cancers. Their tissue‐specific expression and functional versatility make them important regulators in disease biology. Exosomes, which are involved in intercellular communication, reportedly contain higher concentrations of lncRNAs than other vesicles such as apoptotic bodies and microvesicles.^[^ ^23^ ^]^ This enrichment of lncRNAs in exosomes suggests their significant role in mediating tumorigenic processes. Increasing evidence indicates that circulating exosome‐derived lncRNAs serve as valuable biomarkers for cancer detection and prognosis.

#### 2.2.2 Clinical Utility of Exosomal lncRNA for the Diagnosis of GI Cancer

Various studies have demonstrated the significance of exosomal lncRNAs as biomarkers for GI cancer (**Table** **2**). In one of the recent studies, the authors found that serum exosomal lncRNA H19 levels were highly upregulated in GC patients and decreased post‐surgery, also correlating with TNM stage. With an AUC‐ROC of 0.849, exosomal lncRNA H19 demonstrated superior diagnostic performance compared to traditional serum antigens such as CA19‐9 and CA72‐4.^[^ ^24^ ^]^ Similarly, Wei et al. identified NR038975 as a functional lncRNA, significantly upregulated in GC, correlating with LNM and TNM stage. Mechanistically, NR038975 interacts with the NF90/NF45 complex, influencing GC cell proliferation, migration, and invasion. This lncRNA was found to be upregulated in plasma samples (AUC‐ROC = 0.702; *p* < 0.001) obtained from GC patients, as well as in exosomes isolated from plasma (AUC‐ROC = 0.715; *p* < 0.050), suggesting its potential role as a diagnostic marker and therapeutic target for GC.^[^ ^25^ ^]^ Xu et al. found that serum exosomal MIAT levels were significantly higher in GC patients compared to gastric adenoma patients (*p* < 0.001) and healthy individuals (*p* < 0.001). Elevated serum exosomal MIAT also correlated with aggressive clinical features such as poor differentiation (*p* = 0.0264), lymphatic metastasis (*p* = 0.0006), and advanced TNM stage (*p* < 0.0001). Additionally, high serum exosomal MIAT predicted shorter overall (*p* = 0.022) and recurrence‐free survival (*p* = 0.002), and served as an independent prognostic factor for GC (HR = 3.46; *p* = 0.007).^[^ ^26^ ^]^ In contrast, lnc‐GNAQ‐6:1 was significantly under‐expressed in GC patients (*p* = 0.0010). By analyzing serum exosomes from 43 GC patients and 27 healthy subjects, the AUC‐ROC of exosomal lnc‐GNAQ‐6:1 was measured to be 0.736, demonstrating higher sensitivity and specificity for distinguishing GC patients from healthy controls compared to traditional serum markers such as CEA, CA19‐9, and CA72‐4.^[^ ^27^ ^]^

**Table 2:** Summary of the clinical studies that utilized exosomal lncRNAs as a biomarker for gastrointestinal (GI) cancer.

Patients	Specimen	Exosome isolation	RNA extraction	lncRNAs	Profiling	Clinical outcomes	Refs.
81 GC (gastric cancer) 78 HD: healthy donor (HD)	Serum	ExoQuick	miRNeasy Micro Kit	lncRNA H19	Quantitative reverse transcription polymerase chain reaction (PCR)	Higher levels of exosomal lncRNA H19 found in GC patients and correlate with TNM stages	[24]
20 GC 20 HD	Plasma	Not provided	TRIzol	lncRNA NR038975	PCR	Higher levels of exosomal lncRNA NR038975 found in GC patients with AUC‐ROC of 0.715	[25]
109 GC 48 GA (gastric adenoma) 50 HD	Serum	ExoQuick	mirVana miRNA Isolation Kit	lncRNA MIAT	PCR	Higher levels of exosomal lncRNA MIAT indicating higher susceptibility to GC in GA patients with worse clinical outcomes	[26]
43 GC 27 HD	Serum	Exosome extraction kit (details not given)	Exosome RNA extraction kit (details not given)	lnc‐GNAQ‐6:1	PCR	Lower expression of exosomal lnc‐GNA‐6:1 in GC patients compared to HD with AUC‐ROC of 0.736	[27]
51 GC 60 HD	Plasma	Membrane filtration + ultracentrifugation (UC)	RNeasy Mini Kit	79 mRNA and lncRNA upregulated in GC (lncUEGC1 and lncUEGC2)	Exosomal RNA seq, PCR	Higher levels of exosomal lncUEGC1 and lncUEGC2 in stage I and II GC patients	[28]
522 GC 85 GPL (gastric precancerous lesions) 219 HD	Serum	Membrane filtration + UC	RNeasy Kit	lncRNA‐GC1	PCR	Higher levels of exosomal lncRNA‐GC1 in GC patients	[30]

**Figure 2:**
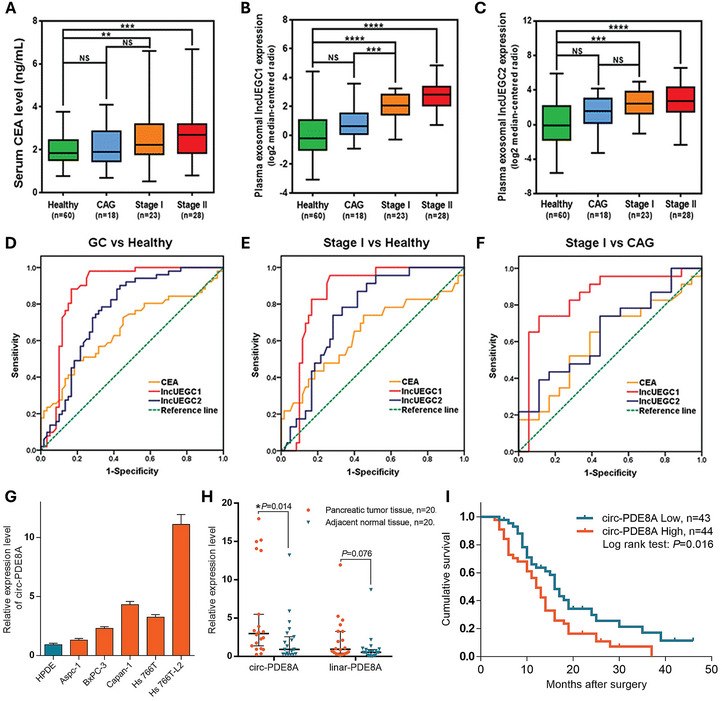
Exosomal nucleic acid as a diagnostic biomarker for gastrointestinal (GI) cancer. A–C) Serum CEA, plasma exosomal lncUEGC1, and plasma exosomal lncUEGC2 levels in CAG, stage I GC, stage II gastric cancer (GC) patients, and healthy controls. D–F) ROC curve analysis of serum CEA, plasma exosomal lncUEGC1, and plasma exosomal lncUEGC2 levels for distinguishing low‐stage GC patients from healthy controls. Differences with *p* < 0.05 were considered statistically significant. Reproduced with permission.^[^ ^28^ ^]^ Copyright 2018, Springer Nature. G) Relative expressions of circ‐PDE8A in pancreatic ductal adenocarcinoma (PDAC) cell lines. H) Relative mRNA expressions of circ‐PDE8A or linear‐PDE8A in 20 pairs of PDAC tumor and adjacent normal tissues. I) Kaplan–Meier survival analysis of 87 PDAC patients according to the relative expression of circ‐PDE8A. Reproduced with permission.^[^ ^34^ ^]^ Copyright 2018, Elsevier. NS, not significant; ^**^ *p* < 0.01; ^***^ *p* < 0.001; ^****^ *p* < 0.0001.

A study using RNA sequencing on plasma exosomes of stage I GC patients (*n* = 10) and healthy individuals (*n* = 5) revealed 79 exosomal lncRNAs and mRNAs upregulated over twofold in GC patients (*q* < 0.00001). The study identified two novel EGC‐specific exosomal lncRNAs, lncUEGC1, and lncUEGC2, which were highly upregulated in stage I and II GC patients (*n* = 51) compared to healthy controls (*n* = 60) (fold change > 5, *p* < 0.0001). ROC curve analysis revealed lncUEGC1 had an AUC of 0.8760 for distinguishing early stage GC patients from healthy controls, indicating its potential as a diagnostic marker for early stage GC (**Figure** **2**A–F).^[^ ^28^ ^]^

lncRNA microarray analysis was also utilized to investigate exosomal RNA markers for GC diagnosis. Zheng et al. identified 199 exosomal lncRNAs with significantly higher expression in GC cells compared to control groups, with lnc‐SLC2A12‐10:1 being notably upregulated. This lncRNA was also upregulated in patient‐derived serum exosomes, showing an AUC‐ROC of 0.776 for discriminating GC patients, which outperformed traditional biomarkers such as CEA, CA19‐9, and CA72‐4. Additionally, exosomal lnc‐SLC2A12‐10:1 expression levels correlated notably with tumor size, TNM stage, LNM, and degree of differentiation.^[^ ^29^ ^]^

The clinical utility of exosomal lncRNAs is now being investigated in large cohorts. Guo et al. collected samples from 522 GC patients, 85 patients with gastric precancerous lesions, and 219 healthy donors (HDs) and found that circulating exosomal lncRNA‐GC1 is a highly effective biomarker for the early detection and monitoring of GC, with an AUC‐ROC of 0.9033. This exosomal lncRNA outperformed conventional serum biomarkers (CEA, CA72‐4, and CA19‐9) for diagnosing GC. The levels of lncRNA‐GC1 were also strongly correlated with disease stages, showing significant differences between successive stages up to stage III. The authors suggest that combining lncRNA‐GC1 detection with conventional endoscopy could substantially improve the early diagnostic rate of GC.^[^ ^30^ ^]^

Furthermore, the function of various exosomal lncRNAs in the progression of GC has been investigated along with their expression in human samples. Pan et al. found that ZFAS1 expression was elevated in GC cells, tumor tissues, and serum, and was associated with lymphatic metastasis and TNM stage. ZFAS1 knockdown inhibited GC cell proliferation and migration by affecting cell cycle progression, inducing apoptosis, and inhibiting epithelial‐to‐mesenchymal transition (EMT), while overexpression had the opposite effects. This lncRNA was found to be elevated in serum exosomes from GC patients, and the delivery of ZFAS1 via exosomes promotes GC progression.^[^ ^31^ ^]^

#### 2.2.3 Limitations and Prospects of Utilizing Exosomal lncRNA as a Biomarker for GI Cancer

Exosomal lncRNAs present distinct advantages as biomarkers for GI cancers due to their involvement in a wide range of cellular processes. LncRNAs regulate gene expression at multiple levels, including chromatin remodeling, transcriptional control, and post‐transcriptional modifications, allowing them to influence numerous pathways involved in tumorigenesis. Unlike miRNAs, which predominantly act by silencing target mRNAs, lncRNAs can act as molecular scaffolds, decoys, or enhancers, providing a broader functional scope in cancer biology. This diverse set of mechanisms allows lncRNAs to modulate complex tumorigenic processes more comprehensively than miRNAs, making them valuable for identifying specific cancer subtypes and stages. Their functional diversity not only aids in tumor characterization but also offers insights into cancer progression, metastasis, and therapeutic resistance.^[^ ^32^ ^]^

However, despite these advantages, several challenges limit the use of exosomal lncRNAs as clinical biomarkers for GI cancer. One of the key challenges is the low abundance of exosomal lncRNAs in body fluids. Unlike smaller RNA species such as miRNAs, lncRNAs are typically present in lower concentrations, making their detection and quantification challenging. Current detection methods, such as qPCR and RNA sequencing, often require extensive sample preparation and highly sensitive technologies to isolate and measure these molecules accurately. The lack of standardized protocols for exosomal lncRNA isolation and analysis further complicates their clinical use. Many existing techniques are optimized for small RNAs, and developing specific methods for the efficient capture and amplification of lncRNAs remains a significant hurdle.^[^ ^33^ ^]^

In addition, the heterogeneity of exosomal lncRNAs presents another limitation. LncRNAs can vary significantly between patients, tumor types, and even stages of cancer progression, which complicates the identification of universally applicable biomarkers. Moreover, lncRNAs often have multiple overlapping functions, acting as molecular decoys, scaffolds, or guides, which can influence a broad range of cellular processes. This functional diversity, while valuable for understanding complex tumor biology, complicates the task of choosing specific lncRNAs as diagnostic biomarkers for GI cancers.

Despite these challenges, advances in RNA amplification techniques and NGS hold promise for overcoming these limitations. Technologies such as digital droplet PCR (ddPCR) and single‐cell RNA sequencing (scRNA‐seq) could improve the sensitivity and accuracy of exosomal lncRNA detection, enabling more precise profiling of these biomarkers. Furthermore, the use of machine learning algorithms or AI technologies may facilitate the analysis of large datasets of exosomal lncRNA profiles. These techniques could help identify patterns and signatures associated with specific cancer types or stages, potentially enabling earlier and more accurate detection of GI cancers. AI‐based approaches could also assist in personalizing treatment set‐ups by correlating exosomal lncRNA profiles with patient responses to specific therapies.

In conclusion, while the utilization of exosomal lncRNAs as biomarkers for GI cancers has shown potential, significant improvements in detection methods, standardization, and data analysis are required before these molecules can be widely adopted in clinical settings. Ongoing advancements across various scientific fields may enable exosomal lncRNAs as reliable diagnostic biomarkers for GI cancer and facilitate personalized treatment, enhancing patients outcomes.

### 2.3 Exosomal circRNAs

#### 2.3.1 Exosomal circRNAs as a Diagnostic Biomarker for GI Cancer

CircRNAs are a unique class of endogenous non‐coding RNAs characterized by their covalent loop structure, which distinguishes them from linear RNAs.^[^ ^35^ ^]^ This circular configuration arises through a back‐splicing event, in which the 3′ and 5′ ends of the RNA molecule are covalently joined, making circRNAs inherently resistant to exonuclease degradation. As a result, circRNAs are exceptionally stable compared to other RNA types (e.g., lncRNAs and miRNAs), which are more susceptible to enzymatic degradation. This stability is a key advantage of circRNAs, particularly in liquid biopsy applications, where reliable and durable biomarkers are essential for monitoring diseases such as GI cancers.

Exosomal circRNAs, in particular, have garnered significant attention as potential diagnostic and predictive biomarkers for GI cancers. Exosomes carry circRNAs in a protected environment, shielding their degradation in circulation. Encapsulation within exosomes further enhances the stability of circRNAs, allowing them to persist in bodily fluids. These properties makes exosomal circRNAs highly attractive for noninvasive diagnostic applications. Recent studies have demonstrated that specific exosomal circRNAs are differentially expressed in cancer patients compared to healthy individuals, with some correlating with tumor progression, metastasis, and prognosis in GI cancer. Furthermore, their resistance to enzymatic degradation ensures that they remain intact throughout the process of sample collection, transport, and storage, enhancing the reliability of diagnostic tests. Due to their tissue‐specific expression and high conservation, exosomal circRNAs have the potential to serve as highly specific biomarkers, providing insights into tumor origin and stage, while offering an effective means for early detection.^[^ ^36^ ^]^

#### 2.3.2 Clinical Utility of Exosomal circRNA for the Diagnosis of GI Cancer

Exosomal circRNAs have been extensively explored as a biomarker for various types of GI cancer (**Table** **3**). Based on RNA sequencing of 8 GC tissues and adjacent normal tissues, Xie et al. discovered a total of 1,445 distinct circRNA candidates, with circSHKBP1 (hsa_circ_0000936) being one of the most differentially expressed RNAs in GC.^[^ ^37^ ^]^ In vitro and in vivo studies demonstrated that circSHKBP1 overexpression was associated with enhanced GC cell proliferation, migration, invasion, and angiogenesis whereas its suppression had the opposite effects. Mechanistically, circSHKBP1 promoted GC progression by sponging miR‐582‐3p to increase HUR expression and VEGF mRNA stability, and by inhibiting HSP90 ubiquitination. The authors found that circSHKBP1 was significantly upregulated in serum exosomes obtained from GC patients compared to HDs, with levels in exosomes being approximately six times higher than in tumors. Additionally, circSHKBP1 levels in exosomes decrease significantly after tumor removal, indicating its origin in GC tissues and suggesting its potential as a biomarker for GC, particularly in advanced stages and poor prognosis.^[^ ^37^ ^]^

**Table 3:** Summary of the clinical studies that utilized exosomal circRNAs as a biomarker for gastrointestinal (GI) cancer.

Patients	Specimen	Exosome isolation	RNA extraction	circRNAs	Profiling	Clinical outcomes	Refs.
20 GC (gastric cancer) 20 HD	Blood	ExoQuick	TRIzol reagent, PARIS Kit, Magnetic RNA‐protein Pull‐down Kit	circSHKBP1	Quantitative reverse transcription polymerase chain reaction (PCR)	Higher levels of circSHKBP1 associated with advanced TNM stages, vascular invasion, and poor prognosis in GC	[37]
64GC 64HD	Plasma	exoEasy Maxi Kit	TRIzol reagent	circRELL1	PCR	Reduced expression of exosomal circRELL1 associated with advanced TNM stage and poor prognosis in GC	[38]
30 GC 30 HD	Plasma	Membrane filtration + ultracentrifugation (UC)	TRIzol reagent	circ‐RanGAP1	PCR	Higher levels of circ‐RanGAP1 associated with an advanced TNM stage, lymph node metastases, and worse survival	[39]
93 PDAC (pancreatic ductal adenocarcinoma)	Plasma	Centrifugation	TRIzol LS	circ‐PDE8A	PCR	Higher levels of circ‐PDE8A associated with tumor progression and shorter overall survival (OS) duration in PDAC	[34]
60 HCC 60 HD	plasma	ExoQuick	exoRNeasy Midi Kit	circ_0051443	PCR	Lower levels of exosomal circ‐051443 found in hepatocellular carcinoma (HCC) patients with an AUC‐ROC of 0.8089	[42]
70 HCC 50 HD	serum	MagCapture Exosome Isolation Kit	RNA simple	circANTXR1	PCR	Higher levels of CircANTXR1 found in HCC patients with an AUC‐ROC of 0.76	[44]
50 HCC 50 HD	Serum	Gradient Centrifugation + UC	TRIzol	circ‐0072088	PCR	Higher levels of Circ‐0072088 found in HCC patients with an AUC‐ROC of 0.899	[41]
82 HCC 47 HD	Serum	ExoQuick	SeraMir Exosome RNA Amplification Kit	circPTGR1	PCR	Exosomes from metastatic cells with a high abundance of circPTGR1 inhibit miR449a‐MET interactions, promoting HCC progression.	[45]
39 HCC	Plasma	Centrifugation + UC	TRIzol	circRNA‐100338	PCR	Higher levels of circRNA‐100338 associated with mTOR signaling pathway and poor prognosis	[46]
112 CRC 28 polyps 74 GC 18 BRCA (breast invasive carcinoma) 24 BLCA (bladder urothelial carcinoma) 32 CESC (cervical squamous cell carcinoma and endocervical adenocarcinoma) 19 KRIC (kidney renal clear cell carcinoma) 42 LUAD (lung adenocarcinoma) 60 HD	Plasma	ExoQuick Plasma Prep with Thrombin Kit	circRNA pull‐down assay, Magna RIP kit	circLPAR1	PCR	Lower levels of exosomal circLPAR1 in CRC patients with an AUC‐ROC of 0.858	[47]
20 CRC 20 HD	Serum	ExoQuick	TriZol	circHIPK3	PCR	Higher levels of exosomal circHIPK3 in CRC patients with an AUC‐ROC of 0.771	[43]
221 CRC 221 HD	Serum	ExoQuick	miRNeasy Serum/Serum Kit	circ‐PNN	PCR	Higher levels of circ‐PNN in CRC patients with an AUC‐ROC of 0.855	[48]
25 CRC	plasma	Sequential differential centrifugation + UC	TRIzol reagent	circ‐133	PCR	Higher levels of exosomal circ‐133 associated with disease progression and metastasis in CRC	[49]
62 CRC 62 HD	plasma	Centrifugation + UC	TRIzol LS reagent	circFMN2	PCR	Higher levels of circFMN2 associated with poor clinicopathological characteristics in CRC	[50]

In another study, circRELL1, which is significantly downregulated in GC tissues, was investigated as a potential biomarker for GC. CircRELL1 was found to inhibit GC cell proliferation, invasion, migration, and anti‐apoptosis both in vitro and in vivo.^[^ ^38^ ^]^ Mechanistically, circRELL1 functions by sponging miR‐637, which indirectly upregulates EPHB3 expression through modulating autophagy. CircRELL1 was found to be transmitted via exosomes, where it suppresses malignant behavior in GC. As a result, low plasma exosomal circRELL1 levels indicated GC with an AUC‐ROC of 0.731, and were associated with worse tumor grade, tumor stage, clinical grade, and lymphatic invasion. Additionally, plasma exosomal circRELL1 levels significantly increased after gastrectomy, implying that these exosomal circRELL1 molecules are shed from normal tissues. Circ‐RanGAP1, which promotes GC invasion and metastasis through the miR‐877‐3p/VEGFA axis, has also been utilized as a biomarker for GC.^[^ ^39^ ^]^ This circRNA has been found to be upregulated in plasma exosomes obtained from GC patients and was strongly associated with advanced TNM stages and increased mortality in GC.

circRNAs have also been investigated as biomarkers for other types of GI cancer. Circ‐PDE8A has been identified to promote PDAC cell invasion by acting as a ceRNA for miR‐338, regulating MACC1, and stimulating growth via the MACC/MET/ERK or AKT pathways. This circRNA was upregulated in plasma exosomes obtained from PDAC patients, showing a strong correlation with lymphatic invasion, advanced TNM stage, and poor survival (Figure 2G,H).^[^ ^34^ ^]^

The diagnostic potential of exosomal circRNAs has also been explored in HCC. Specifically, circ‐0004277 is significantly upregulated in HCC cells, where its overexpression enhances HCC proliferation, migration, and EMT by inhibiting ZO‐1. Exosomal circ‐0004277 from HCC cells also stimulated EMT in peripheral cells through cellular communication, further promoting HCC invasion into surrounding normal tissues. Consequently, exosomal circ‐0004277 was overexpressed in HCC patients, exhibiting an AUC of 0.816 with a sensitivity of 58.3% and specificity of 96.7%.^[^ ^40^ ^]^ Likewise, exosomal circANTXR1 and circ‐0072088, which were found to be overexpressed in HCC cells, were also elevated in serum samples obtained from HCC patients. Exosomal circANTXR1 and circ‐0072088 demonstrated high diagnostic capabilities with AUC‐ROC of 0.76 and 0.89, respectively, for distinguishing HCC patients from HDs. In the case of exosomal circ‐0072088, overexpression of this marker was strongly associated with a low 5‐year survival rate.^[^ ^41^ ^]^ In contrast, exosomal circ‐0051443, which is transferred from normal cells to HCC cells, plays a major role in suppressing HCC progression. Thus, low levels of exosomal circ‐0051443 were indicative of HCC, with an AUC‐ROC of 0.8089 for distinguishing HCC patients from HDs.^[^ ^42^ ^]^ Similarly, exosomal circRNA‐100338, circRNA‐SORE, and circPGR1 have also been investigated as diagnostic and prognostic biomarkers for HCC.^[^ ^45^, ^46^ ^]^

For CRC, plasma exosomal circLPAR1 has been identified as a CRC‐specific biomarker via RNA sequencing, exoRBase database analysis, and tissue microarray. This circRNA was notably downregulated during CRC development, which increased upon tumor resection. The diagnostic capability of circLPAR1 was investigated, showing AUC‐ROC of 0.858, which was superior to conventional serum biomarkers (CEA and CA19‐9).^[^ ^47^ ^]^ The expressions of serum exosomal circ‐PNN and circHIPK3 were also elevated in CRC patients. The AUC‐ROC of these exosomal circRNAs were 0.855 and 0.771 for discriminating patients with CRC from HDs, respectively, demonstrating their potential as CRC‐specific biomarker.^[^ ^37^, ^43^ ^]^ Likewise, other exosomal circRNAs such as circFMN2 and circ‐133 have been observed to be highly correlated with malignancies in colorectal.^[^ ^49^, ^50^ ^]^

#### 2.3.3 Limitations and Prospects of Utilizing Exosomal circRNA as a Biomarker for GI Cancer

As aforementioned, exosomal circRNAs offer unique advantages over exosomal miRNAs and lncRNAs as potential biomarkers for GI cancers. Unlike linear RNAs, circRNAs have a covalently closed loop structure, which makes them highly resistant to exonuclease‐mediated degradation, resulting in exceptional stability in circulation. This stability surpasses that of both miRNAs and lncRNAs, enhancing their potential as robust biomarkers. Exosomal circRNAs also have the ability to act as miRNA sponges, regulating miRNA activity and influencing gene expression indirectly. This unique mechanism, along with their tissue‐specific expression, makes circRNAs promising candidates for early cancer detection and prognosis.

However, challenges remain in the efficient isolation and characterization of exosomal circRNAs, as current methods are often not optimized for their unique circular structure. To address these challenges, the development of circRNA‐specific isolation techniques and the refinement of RNA sequencing technologies tailored to circular RNA detection are required. Additionally, the integration of circRNAs into multiomics studies could help decipher their complex roles in cancer biology. Future works involve using various data interpretation models to integrate exosomal circRNA profiles with miRNA and lncRNA data, potentially uncovering new regulatory networks that contribute to cancer progression. This combined approach may enable more precise diagnostic tools and personalized therapeutic strategies for GI cancer, leveraging the complementary roles of exosomal miRNAs, lncRNAs, and circRNAs.

### 2.4 Exosomal Proteins

#### 2.4.1 Exosomal Proteins as a Diagnostic Biomarker for GI Cancer

In addition to nucleic acids, exosomal proteins may also function as biomarkers for the diagnosis and prognosis of GI cancer. These proteins include a diverse array of molecules, such as membrane proteins, signaling molecules, enzymes, and heat shock proteins. Exosomal proteins are reflective of the cell of origin, making them valuable for identifying cancer‐specific molecular signatures. In the context of GI cancers, exosomal proteins contribute to various aspects of tumor biology, including tumor growth, immune evasion, and metastasis. For example, exosomal proteins can modulate the tumor microenvironment by transferring oncogenic signals between tumor cells and stromal cells, promoting angiogenesis and invasion. Additionally, the presence of specific exosomal proteins has been correlated with disease progression, offering potential for early detection and monitoring therapeutic responses.^[^ ^51^ ^]^

Exosomal proteins are classified into two categories. The first type includes common exosomal proteins such as heat shock proteins (HSP), integration‐related proteins, transmembrane transport proteins and tetraspanins (e.g., CD9 and CD63).^[^ ^52^ ^]^ The second type includes proteins present in specific types of exosomes, mainly depending on their cell of origin. For example, TRIM3 (Tripartite motif 3) (serum, cell lines), GKN1 (Gastrokine‑1) (tissue), TGF‐β1 (Transforming growth factor‑beta 1) (cell line), CD97 (cell line), HSP‐60 & HSP‐70 (malignant ascites).^[^ ^53^ ^]^ Notably, scientists are increasingly focusing on the second type of exosomal proteins that may be specifically derived from GI tissues.

#### 2.4.2 Clinical Utility of Exosomal Proteins for the Diagnosis of GI Cancer

**Figure 3:**
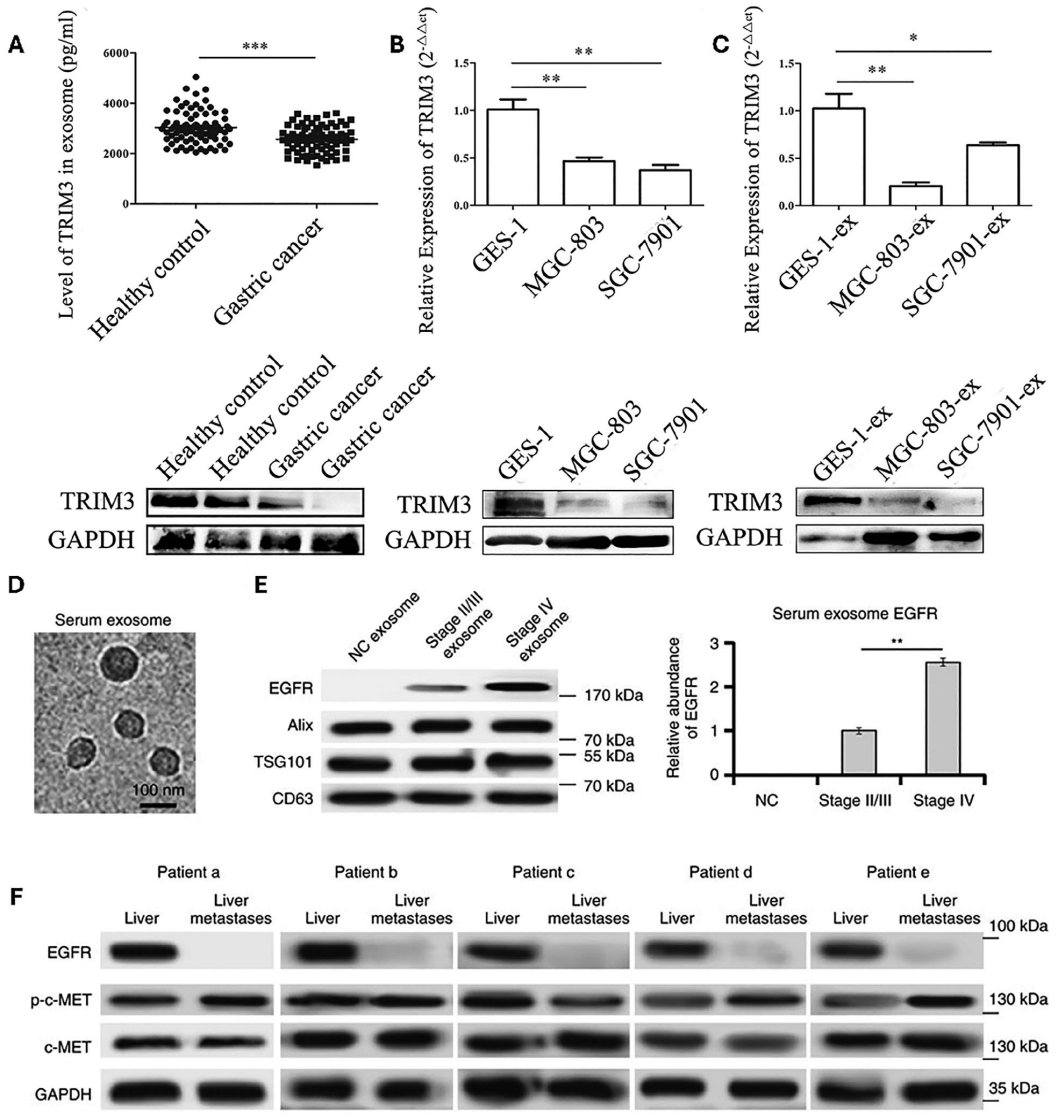
Exosomal proteins as a diagnostic biomarker for gastrointestinal (GI) cancer: A) Exosomal TRIM3 levels in serum of gastric cancer patients and healthy controls (^***^ *p* < 0.001). B) Relative expression of TRIM3 genes and proteins in gastric epithelial cell lines and gastric cancer cell lines (^**^ *p* < 0.01). C) Relative expression of TRIM3 genes and proteins in exosomes obtained from gastric epithelial cell lines and gastric cancer cell lines (^**^ *p* < 0.01). Reproduced with permission.^[^ ^55^ ^]^ Copyright 2018, Springer Nature. D) Electron microscope images of exosomes isolated from human serum. E) Relative EGFR expression levels in exosomes obtained from serum of healthy donors (HDs), stage II/III GC patients, and stage IV gastric cancer (GC) patients. F) Western blot analysis of EGFR, c‐MET, and p‐c‐MET in para‐carcinoma tissue (liver) and GC liver metastases. Reproduced with permission.^[^ ^63^ ^]^ Copyright 2017, Springer Nature.

As previously mentioned, various exosomal proteins are being investigated as biomarkers for GI cancer (**Table** **4**). Located on chromosome 11p15.5, TRIM3 is a member of the TRIM family, characterized by a coiled‐coil domain, a RING domain, and one or two B‐boxes.^[^ ^54^ ^]^ The upregulation of TRIM3 is known to inhibit tumor cell proliferation and metastasis. Fu et al. validated the function of exosomal TRIM3, demonstrating that exosomes from TRIM3‐overexpressing cell lines inhibited GC growth and reduced metastatic potential by regulating stem cell factors and EMT regulators. Further proteomic analyses of exosomes from the serum of GC patients revealed a total of 33 proteins that show significant differences between GC patients and HDs, with TRIM3 being one of the downregulated markers. ELISA and Western blot analyses supported that exosomes obtained from the serum of GC patients exhibited significantly lower TRIM3 expression compared to those from HDs (*p* < 0.001) (**Figure** **3**A–C).^[^ ^55^ ^]^ These findings suggest that low TRIM3 expression in serum exosomes can serve as a biomarker for GC diagnosis, and the delivery of TRIM3 via exosomes may offer a new approach for GC therapy.

**Table 4:** Summary of the clinical studies that utilized exosomal proteins as a biomarker for gastrointestinal (GI) cancer.

Patients	Specimen	Exosome isolation	Proteins	Quantification	Clinical outcomes	Refs.
20 gastric cancer (GC) 20 HD	Serum	ExoQuick	Tripartite motif containing 3 (TRIM3)	Enzyme‐linked immunosorbent assay (ELISA) Western blot (WB)	Lower levels of exosomal TRIM3 found in GC patients	[55]
61 GC	Plasma	Ultracentrifugation (UC)	Transforming growth factor‐β1 (TGF‐β1)	ELISA	Higher levels of exosomal TGF‐β1 associated with a high TNM stage, as well as LN metastasis in GC	[61]
20 GC 20 HD 5 with liver metastasis	Serum	Exosome Isolation Kit (Details not provided)	Epidermal growth factor receptor (EGFR)	WB	Higher levels of exosomal EGFR associated with a high TNM stage in GC	[63]
4 cagA+ H. pylori 1 HD	Serum	UC	Cytotoxin associated gene A (CagA)	Micro BCA (bicinchoninate) assay	CagA‐containing exosomes having potential to promote the development of extragastric disorders	[72]
238 GC	Serum	ExoQuick	Human epidermal growth factor receptor 2 (HER2)	ELISA WB	HER2 expressing exosomes derived from patients’ serum serve as a stable prognosis biomarker for assessing gastric cancer	[73]
15 PCa (prostate cancer) 15 BPH (benign prostatic hyperplasia) 15 HD	Plasma	UC	Prostate‐specific antigen (PSA)	ELISA	CD81/PSA containing exosomes level are four‐times higher in PCa patients than other donors	[66]
102 colorectal cancer (CRC) 80 HD	Plasma	ExoCapTM	Glypican 1 (GPC1)	WB	GPC1 positive exosomes originated from CRC patients’ plasma are 10‐fold higher than healthy donor	[68]
194 CRC 191 HD	Serum	ExoScreen	CD147	ELISA	CD147‐expressing CD9+ exosomes are present in higher quantities in CRC patient sera compared to healthy donors (HDs)	[67]

CGKN1, alternatively referred to as antral mucosal protein (AMP)‐18, is a GKN family member situated on chromosome 2p13.3.^[^ ^56^ ^]^ GKN1 is secreted by gastric epithelial cells, initially stored in cytoplasmic granules, and then transported into the extracellular environment. The majority of GKN1 is transported via exosomes, as evidenced by the minimal differences in GKN1 protein expression levels between whole serum and serum exosomes (3.58 ± 0.54 ng mL^−1^ vs. 3.28 ± 0.82 ng mL^−1^). It has been found that the absorption of exosomal GKN1 by stomach epithelium may inhibit the proliferation of GC cells, potentially representing a crucial self‐protective mechanism of the body in response to GC.^[^ ^57^ ^]^ As demonstrated by Guo et al., this protein is expressed at low levels in tumors but is highly expressed in adjacent normal tissue.^[^ ^58^ ^]^ Specifically, the concentrations of serum GKN1 in 100 HDs and 150 patients with GC were significantly different, at 6.35 ± 0.82 ng mL^−1^ and 3.50 ± 0.57 ng mL^−1^, respectively (*p* < 0.0001). The low serum GKN1 expression was specific to GC, as patients with other types of tumors, such as HCC (6.26 ± 0.95 ng mL^−1^) or CRC (6.07 ± 0.92 ng mL^−1^), had serum GKN1 levels similar to those of HDs.^[^ ^57^ ^]^ These findings suggest that low exosomal GKN1 expression could be a potential indicator of GC.

TGF‐β1 is a homodimeric polypeptide with a molecular weight of 25 kDa, released by both tumor and immune cells. Multiple studies have demonstrated that TGF‐β1 plays a dual role in tumor development. During the early stages of tumor formation, TGF‐β1 inhibits cellular proliferation and induces apoptosis. TGF‐β1 has been implicated in NK cell damage, metastasis, and the recurrence of GC. The role of TGF‐β1 and its receptors in GC progression has been confirmed by various in vitro and in vivo studies. For instance, knockdown of TGF‐β1 or its receptors on gastric cancer cell lines inhibited cell migration, invasion, and proliferation.^[^ ^59^ ^]^ Several studies have explored the potential of serum exosomal TGF‐β1 as a predictor of GC progression. Serum exosomal TGF‐β1 levels were found to be elevated in patients with GC, and this elevation was positively correlated with tumor TNM stage.^[^ ^60^ ^]^ In another study using ELISA, Yen et al. measured exosomal TGF‐β1 levels in the gastroepiploic veins of 61 patients with stomach cancer.^[^ ^61^ ^]^ Their findings revealed that exosomal TGF‐β1 expression was higher in patients with stages II, III, and IV GC compared to those with stage I. Furthermore, patients with LNM exhibited twice the level of exosomal TGF‐β1 expression as those without LNM, suggesting that exosomal TGF‐β1 expression can be an indicator of high malignancies in GC.

Human epidermal growth factor receptor 1 (HER1) which is also well known as EGFR belongs to the HER (ErbB) family. EGFR is a transmembrane glycoprotein and, like other members of the ErbB family, belongs to the receptor tyrosine kinase group. It comprises the following: an extracellular domain, a lipophilic transmembrane region, an intracellular domain containing tyrosine kinase, and a carboxy‐terminal region.^[^ ^62^ ^]^ This protein is recognized as one of the most established signaling pathways in cancer progression and has been used as a target receptor for isolating various cancer‐specific biomarkers, including exosomes. Exosomal EGFR expression was revealed to be associated with GC progression from a myriad of study. In a study conducted by Zhang et al., EGFR was found to be enriched in the serum exosomes of GC patients, whereas its expression was significantly lower in HDs. Additionally, the concentration of exosomal EGFR was higher in the serum of stage IV GC patients compared to those in lower stages, demonstrating the potential of exosomal EGFR as a prognostic biomarker of GC (Figure 3D–F).^[^ ^63^ ^]^

ApoE, also known as Apolipoprotein, is a 39 kDa small secretory glycoprotein found in chromosome 19q13.2. The correlation between exosomal ApoE and GC progression has been confirmed from various studies. For instance, Zheng et al. demonstrated that exosomes originating from M2 macrophages exhibited a distinct selectivity for ApoE protein.^[^ ^64^ ^]^ The delivery of functional ApoE‐expressing exosomes from tumor‐associated macrophages (TAMs) to cancer cells may stimulate the PI3K/Akt signaling pathway, hence promoting the metastasis of GC cells. The roles and diagnostic capabilities of exosomal ApoE are thus being extensively investigated for various diseases including GI cancer.^[^ ^65^ ^]^

The expression of other well‐established cancer‐associated proteins such as HER‐2, programmed death ligand 1 (PD‐L1), prostate‐specific antigen (PSA), glypican‐1 (GPC1), and CD147 also have been analyzed in exosomes to utilize these exosomal proteins as a biomarker for GI cancer. Li et al. demonstrated that higher exosomal HER2 levels were associated with better prognosis and survival outcomes in GC patients receiving trastuzumab, an anti‐HER2 antibody. The elevated exosomal HER2 levels correlated with improved PFS and OS upon trastuzumab treatment.^[^ ^65^ ^]^ The expression of various immune checkpoint proteins are also being investigated as a biomarker for GI cancer. The elevated exosomal PD‐L1 in preoperative plasma was associated with significantly worse OS in GC patients and served as an independent prognostic factor. Interestingly, exosomal PD‐L1 levels were linked to an immunosuppressive environment, correlating negatively with the numbers of CD4+ and CD8+ T cells, as well as granzyme B levels, showing a stronger association than that observed with soluble PD‐L1 expression. One of the most common PCa markers, PSA, was overexpressed in the serum exosomes obtained from PCa patients. Microenvironmental acidity has been linked to the release of exosomes containing PSA. Elevated levels of these PSA‐expressing exosomes were observed in the blood of PCa patients but not in those with benign prostatic hypertrophy (BPH) or HDs, revealing potential of exosomal PSA as a diagnostic biomarker for PCa.^[^ ^66^ ^]^ Similarly, high levels of exosomal CD147 were detected in CRC patients, with its expression decreasing after surgery.^[^ ^67^ ^]^

Upregulated glycosylation is also a prominent biomarker for cancer diagnosis. Cancer cells exploit cellular glycosylation in various ways, including the development of cancerous traits, proliferation, immune evasion, and enhanced drug resistance.^[^ ^68^ ^]^ The selective detection of glycans or the assessment of their expression levels is, therefore, considered a promising approach for diagnosing and predicting the progression of various GI cancers. For example, Choi et al. compared lectin levels on the cell membrane in PCa using fluorescence‐based lectins with a high and specific affinity for sialic acid‐ or fucose‐containing glycans. These lectins were conjugated to bifunctional Janus nanoparticles (JNPs), facilitating interactions with PCa cell‐derived exosomes within a microfluidic device. Statistical analysis of the SNA, AAL, and CA19‐9 antibody groups revealed that fucose was the only biomarker that significantly distinguished the presence of PCa in metastatic plasma samples (*p* < 0.0001, *n* = 3).^[^ ^69^, ^70^ ^]^ The diagnostic potential of GPC1‐expressing exosomes was also validated using plasma samples from CRC patients. GPC1‐expressing exosomes were significantly elevated in the plasma of CRC patients compared to healthy controls, and their levels decreased following surgical treatment.^[^ ^68^ ^]^

#### 2.4.3 Clinical Utility of Exosomal Proteins for the Diagnosis of GI Cancer

Exosomal proteins offer a promising approach to GI cancer biomarkers due to their direct reflection of cellular processes and the tumor microenvironment. These proteins include membrane‐bound molecules, signaling proteins, enzymes, and other cell‐specific markers, which are reflective of the cellular origin and provide real‐time insights into oncogenic pathways and immune evasion mechanisms. Exosomal proteins can reveal specific cancer signatures, particularly when combined with high‐throughput proteomics techniques such as mass spectrometry, enabling the detection of post‐translational modifications that add further depth to cancer profiling. However, despite their potential for detailed molecular analysis, several challenges hinder the clinical application of exosomal proteins as reliable GI cancer biomarkers.^[^ ^71^ ^]^

A major limitation is the heterogeneity of exosomal proteins, which complicates the identification of specific cancer‐associated profiles. Protein contents in exosomes are highly variable and often contain complex mixtures from multiple cell types. This variability makes it challenging to isolate proteins that are both specific to cancer and resistant to interference from noncancerous cells in circulation. Furthermore, exosomal proteins tend to be less stable compared to encapsulated RNAs, as proteins can degrade more readily in the bloodstream, particularly in the absence of consistent stabilization methods.

To overcome these challenges, advancements in proteomics, such as machine learning algorithms applied to mass spectrometry data, could enhance the sensitivity and specificity of exosomal protein detection. In addition, integrating protein biomarkers with RNA markers (miRNAs, lncRNAs, circRNAs) may allow for a multiparametric diagnostic platform that provides both stable genetic information and dynamic protein signals. Future research may also explore methods for selectively isolating exosomes from specific tissue origins, such as cancer‐specific surface markers, to reduce heterogeneity and improve the specificity of exosomal protein biomarkers.

## 3 Exosomes for GI Cancer Therapeutics

### 3.1 Engineering Exosomes as Versatile Therapeutic Platforms

Exosomes, naturally occurring EVs, play essential roles in various physiological and pathological processes by delivering active biomolecules to target cells. As a result, they are extensively investigated as potential therapeutic agents. Exosomes can be sourced from a variety of cells, including milk, mesenchymal stem cells (MSCs), HEK293 cells, dendritic cells (DCs), red blood cells, and cancer cells, depending on the target disease.^[^ ^74^ ^]^ Exosomes carry a diverse array of biomolecules from their parent cells, including nucleic acids, lipids, metabolites, and proteins. Consequently, their function may vary significantly depending on the cell of origin. For example, exosomes derived from activated T lymphocytes have the potential to activate other immune cells, whereas exosomes released from MSCs can exert immunosuppressive effects. Recent studies suggest that exosomes may selectively accumulate specific cellular components through a targeted mechanism, highlighting the importance of the cell of origin in modulating physiological and pathological processes.

Exosomes can be further engineered to encapsulate a wide range of therapeutic molecules, making them promising candidates for drug delivery systems in GI cancer treatment.^[^ ^4^ ^]^ Their high stability, biocompatibility, and target specificity, combined with their capacity to be loaded with diverse chemical compounds, enhance their potential as effective drug delivery vehicles.^[^ ^4^ ^]^ For instance, Agrawal et al. demonstrated that the inhibitory effects of paclitaxel can be significantly enhanced by encapsulating the drug within milk‐derived exosomes for oral delivery.^[^ ^75^ ^]^ The therapeutic efficacy of exosome‐based drug delivery systems is also known to vary depending on their cellular origin. A study comparing the antitumor efficacy of doxorubicin (DOX)‐loaded exosomes revealed that, despite lower drug loading efficiency, macrophage‐derived exosomes exhibited the highest antitumor efficacy among the tested sources, including PCa cells and pancreatic stellate cells.^[^ ^76^ ^]^ These findings suggest that variations in exosomal contents can impact the loading and delivery of therapeutic cargoes, revealing the importance of carefully selecting both the therapeutic molecules and the originating cell type for optimal drug delivery.

In addition to drug loading, the surface of exosomes can be engineered with various organic and inorganic molecules to enhance target specificity or confer additional therapeutic functionalities. Exosomes contain various peptides, tetraspanins, and integrins that selectively target specific cells, providing potential opportunities for developing more precise drug delivery techniques. Their target specificity may be further improved by additional surface modification. Cui et al. modified the exosome surface using a bone‐targeting peptide to effectively deliver siShn3, resulting in targeted osteoblasts and enhanced anti‐osteoporosis effects. In another study, exosomes derived from fibroblast‐like mesenchymal cells were engineered to effectively target Kras^G12D^, outperforming liposomes mainly due to CD47‐mediated protection from phagocytosis and improved cellular uptake through micropinocytosis.^[^ ^77^ ^]^

Artificial exosome‐like hybrid vesicles can also be engineered by incorporating cell membrane proteins from multiple cell types into synthetic phospholipid bilayers. Membrane proteins such as CD47 on the surface of red blood cells are known to inhibit phagocytosis, while MCF‐7 cancer cells express adhesion proteins on their membranes that bind to other cancer cells. The hybridization of these membrane bilayers results in artificial exosome‐like vesicles with enhanced tumor targeting, reduced phagocytosis, and superior antitumor effects compared to conventional liposomes.^[^ ^78^ ^]^

Exosomes can also be modified with inorganic materials to obtain additional functionalities. Qi et al. developed superparamagnetic nanoparticle cluster exosomes (SMNC‐EXOs) by conjugating holo‐transferrin to superparamagnetic nanoparticles. These exosomes demonstrated enhanced targeted drug delivery and tumor suppression in animal models by exhibiting strong magnetic responsiveness and improved cancer targeting when subjected to an external magnetic field.^[^ ^79^ ^]^ Exosomes co‐loaded with sonosensitizers Chlorin e6 (Ce6) and immune adjuvant 8484 (Exo^Ce6+R848^) were developed to effectively deliver to tumor sites and enhance anti‐tumor immunity. Ultrasonic irradiation, applied after the accumulation of exosomes at the tumor site, enhanced dendritic cell maturation and reprogramed macrophages from an immunosuppressive M2‐like phenotype to an anti‐tumor M1‐like phenotype.^[^ ^80^ ^]^

**Figure 4:**
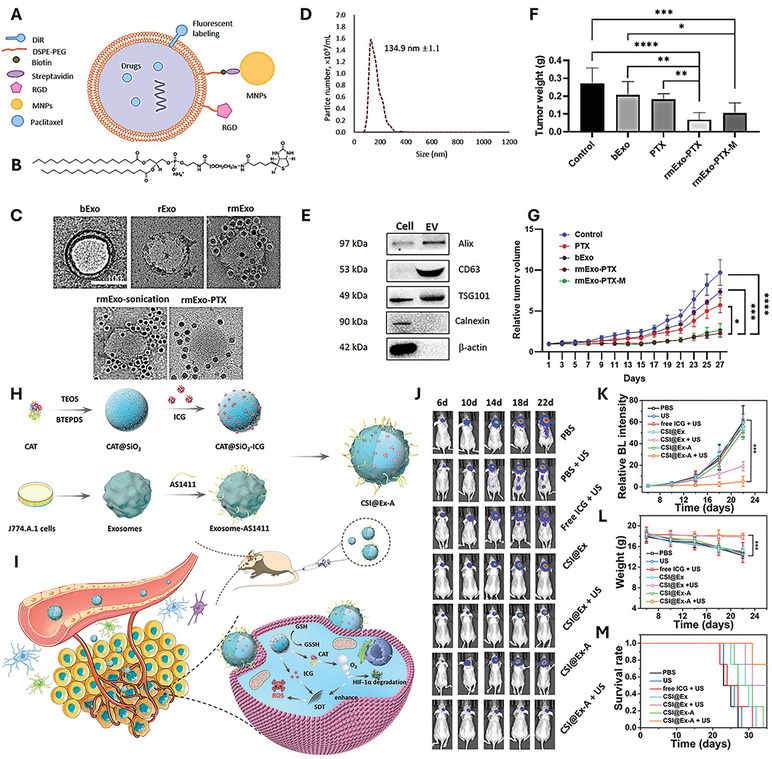
Engineered extracellular vesicles (EVs) for the growth inhibition of gastrointestinal (GI) cancer. A) Engineering of EVs for the efficient delivery of paclitaxel (PTX) via surface modification with RGD peptides and MNPs; B) EVs modified with DSPE‐PEG‐biotin to incorporate streptavidin‐labeled MNPs. C) TEM images of EVs from PANC‐1cells, followed by PTX encapsulation, RGD modification, and MNP decoration. D) Size distribution of EVs. E) Western blot analysis to confirm the expression of EV‐specific and cell‐specific biomarkers in PANC‐1‐derived EVs and cell lysates. F) Excised tumor weights followed by the treatment with the engineered EVs (mean ± SD, ^**^ *p* < 0.01; ^***^ *p* < 0.001). G) Therapeutic efficacy of engineered EVs in PANC‐1 xenograft model (mean ± SEM). Reproduced with permission.^[^ ^89^ ^]^ Copyright 2022, Elsevier. H) Preparation of CSI@Ex‐A: Schematic of the preparation process for CSI@Ex‐A. I) Schematic of biodegradable CSI@Ex‐A for glioblastoma SDT. J) Bioluminescence images of U87‐tumor‐bearing mice upon treatment with CSI@Ex‐A. K) Quantification of fluorescence intensity from bioluminescence images. L) Total weights during treatment (mean ± SD). M) Survival analysis of CSI@Ex‐A treated mice (mean ± SD, ^*^ *p* < 0.05; ^**^ *p* < 0.01; ^***^ *p* < 0.001). Reproduced with permission.^[^ ^81^ ^]^ Copyright 2022, Wiley.

In another study, a biodegradable nanoplatform named CSI is developed by encapsulating catalase (CAT) within silica nanoparticles (CAT@SiO_2_) to alleviate tumor hypoxia, followed by loading these particles with the sonosensitizer indocyanine green (ICG) in macrophage‐derived exosomes. Leveraging the natural ability of macrophages to traverse the blood‐brain barrier (BBB), the surface of CSI‐containing exosomes was further modified with AS1411 aptamers (CSI@Ex‐A). This new exosome‐based nanoplatform successfully released CAT to alleviate hypoxia and improve SDT efficacy in vitro and in vivo, demonstrating a strong potential for clinical translation as a GBM therapeutics (**Figure** **4**H–M).^[^ ^81^ ^]^

Various strategies are now available for loading therapeutic agents into exosomes and enhancing the expression of specific targets, including coincubation, electroporation, and chemical transfection. The efficiency of these engineering processes varies and discovering more effective construction strategies will require further in‐depth research. In the following subsections, we will summarize studies that have engineered exosomes to utilize them as therapeutic agents for GI cancer. These studies are categorized based on the therapeutic functions of exosomes, including growth/metastasis inhibition, reversing drug resistance, and immune modulation.

### 3.2 Exosomes for Inhibiting Growth and Metastasis in GI Cancer

#### 3.2.1 Exosomes as Therapeutic Agents for Growth and Metastasis Inhibition

Exosomes have emerged as potential therapeutic agents for GI cancer treatment due to their ability to inhibit tumor growth and metastasis. As aforementioned, these vesicles can be engineered to deliver therapeutic payloads, such as small RNAs, proteins, or chemotherapeutic drugs, directly to tumor cells, thereby enhancing the efficacy of cancer treatments. For instance, studies have shown that exosomes derived from cancer‐associated fibroblasts or MSCs can be modified to deliver siRNAs targeting oncogenic pathways, subsequently suppressing tumor progression in CRC and GC models.^[^ ^77^ ^]^ These exosome‐based therapies have the advantage of specificity, as exosomes can be loaded with tumor‐targeting ligands or engineered to carry specific therapeutic molecules that act on cancer cells without affecting surrounding healthy tissues.

Beyond drug delivery, exosomes can modulate the tumor microenvironment by regulating processes such as angiogenesis, immune evasion, and cell signaling. For example, exosomes loaded with miRNA inhibitors have been shown to reduce the metastatic potential of GI cancers by disrupting pathways related to EMT, which is critical for cancer metastasis.^[^ ^82^ ^]^ As demonstrated in numerous studies, exosome‐based therapies have the potential to provide a multifaceted approach to treating GI cancers by directly targeting tumor cells and modulating the tumor microenvironment to inhibit growth and metastasis.

#### 3.2.2 Therapeutic Potential of Exosomes for Inhibiting Growth and Metastasis

T‐DM1, or trastuzumab emtansine, is an antibody‐drug conjugate (ADC) that links trastuzumab with emtansine, a tubulin inhibitor. This medication specifically targets HER2‐positive cancer cells, leading to mitotic arrest and apoptosis by releasing the cytotoxic drug emtansine within the cells. In a recent study, Barok et al. observed that T‐DM1 can specifically bind to exosomes derived from HER2‐expressing cancer cells (**Table** **5**). The treatment of these T‐DM1‐containing exosomes on HER2‐expressing cancer cells resulted in growth inhibition and the activation of caspases 3 and/or 7, prompting the apoptosis of the cancer cells.^[^ ^83^ ^]^

**Table 5:** Summary of the exosome‐based therapeutics for inhibiting growth and metastasis of gastrointestinal (GI) cancer.

Exosomes		Encapsulation/loading		Surface modification		Therapeutic outcomes	Refs.
Source	Isolation	Cargoes	Method	Molecules	Method		
Cancer cell lines	Ultracentrifugation (UC)	Trastuzumab emtansine (T‐DM1)	Incubation of the parental cells with T‐DM1	N/A	N/A	T‐DM1‐containing exosomes from HER‐2 positive cancer cells inhibit cell growth and activates caspases 3 and 7	[94]
Macrophage	ExoQuick	miR‐21 inhibitor	Electroporation	N/A	N/A	Exosomes with miR‐21 inhibitors reduce migration of GC cells and induce their apoptosis	[84]
Mesenchymal stromal cells	Total Exosome Isolation Kit	miR‐145‐5p	Exo‐Fect	N/A	N/A	MiR‐145‐5p carrying EVs inhibits pancreatic ductal adenocarcinoma (PDAC) progression and reduce invasive potential by modulating transforming growth factor (TGF) β/Smad pathway	[85]
HEK293T	UC	siHGF	Transfection of parental cells using Lipofectamine	N/A	N/A	HGF siRNA‐loaded exosomes suppress migration and proliferation of tumor cells	[95]
Pancreatic cancer (PC) cells	UC	siPAK4	Electroporation	N/A	N/A	PAK4 knockdown using Exo‐siPAK4 reduced PC tumor growth, enhanced survival in vivo, and induced tumor apoptosis with minimal toxicity	[86]
Mesenchymal stem cells (MSCs)	UC + Filtration	miR‐34a‐5p Si‐c‐MYC	Transfection of parental cells using Lipofectamine	N/A	N/A	MSC‐EVs carrying si‐c‐MYC or miR‐34a‐5p reduced colorectal cancer (CRC) cell invasion and proliferation	[87]
Milk	UC	Oxaliplatin	Electroporation	GE11 peptide	Modification using amphiphilic linkers	GE11 peptide‐conjugated, oxaliplatin‐loaded exosomes enhanced uptake by EGFR‐expressing cancer cells, increasing apoptosis and reducing viability in CRC,	[88]
Monocyte and PCa cell lines	UC	Paclitaxel	Sonication	RGD peptides MNPs	Modification using amphiphilic linkers	RGD‐modified EVs targeted αvβ3‐expressing PCa cells and effectively penetrated and regressed tumors, due to integrin β3‐mediated homing properties.	[89]
HEK293T	UC + Filtration	miR‐26a	Electroporation	Apo‐A1	Parental cells were transfected with ApoCD63 plasmid using Lipofectamine	MiR‐26a‐loaded Apo‐A1‐exosomes inhibit cell migration, proliferation, and growth through miR‐26a‐mediated downregulation of cell cycle regulatory proteins.	[92]
MSCs	UC	anti‐miRNA‐221 nucleotides	Electroporation	iRGD	Parental cells were infected by LV‐iRGD‐Lamp2b	iRGD‐modified exosomes were accumulated on the tumor site while loading of anti‐miR‐221 suppressed tumor growth	[91]
HEK293T	UC	Doxorubicin (DOX)	Incubation	AS1411 (anti‐nucleolin) aptamer	Chemical conjugation	DOX‐loaded, AS1411 aptamer‐functionalized exosomes demonstrated effective tumor growth suppression and long‐term accumulation in nucleolin‐overexpressing CRC through targeted ligand‐receptor interactions.	[96]
MSCs	Filtration + UC	DOX	Electroporation	MUC1 aptamers	Chemical conjugation	MUC1 aptamer‐decorated, DOX‐loaded exosomes significantly enhanced targeted drug delivery and tumor suppression in vivo, showing superior tumor accumulation and clearance compared to free DOX	[90]
A33‐positivie LIM1215 cells	UC	DOX	Incubation	Supermagnetic iron oxide NPs functionalized with anti‐A33 Ab	Incubation (antibodies attached to A33 on exosomes)	A33 antibody‐coated superparamagnetic nanoparticles bound to DOX‐loaded exosomes effectively targeted CRC cells, demonstrating enhanced tumor targeting, antiproliferative effects, reduced cardiotoxicity, and prolonged survival in vivo	[93]

miRNA itself, or its inhibitors, can also be delivered to the target cells via exosomes. In a study conducted by Wang et al., exosomes were used as carriers to deliver a miRNA inhibitor specifically targeting miR‐21, which is known to promote the progression of GC. MiR‐21 inhibitors were encapsulated into macrophage‐derived exosomes via electroporation, and the delivery of these engineered exosomes successfully modulated the growth of gastric cancer cells.^[^ ^84^ ^]^ In another study, miR‐145‐5p mimics were loaded onto exosomes derived from human umbilical cord mesenchymal stromal cells using commercial exosome transfection reagents. MSCs have been utilized as a major source of exosome‐based therapeutics due to their self‐repair, multidirectional differentiation, immune modulation, and homing properties. Further engineering of these MSC‐derived exosomes successfully inhibited proliferation and invasion of PDAC, increased their apoptosis and cell cycle arrest in vitro, and significantly reduced tumor growth in a mouse model by modulating TGF‐β/Smad pathway.^[^ ^85^ ^]^

RNA interference is a promising therapeutic approach due to its ability to selectively silence specific genes with high efficacy. However, the clinical application of siRNA is limited by issues such as non‐specific cellular uptake and susceptibility to nuclease degradation. Exosomes are increasingly being used as delivery vehicles for siRNA in the treatment of GI cancer. For instance, HEK293T cells were transfected with hepatocyte grow factor (HGF) siRNA and therapeutic efficacy of exosomes containing these siRNAs were tested both in vitro and in vivo. Exosomes loaded with HGF siRNA were shown to suppress cancer cell and vascular cell proliferation and migration in a coculture model, and reduce tumor and blood vessel growth, highlighting their potential for targeted GI cancer therapy. Similarly, siRNA targeting P21‐activated kinase 4 (PAK4) was encapsulated in exosomes derived from PC via electroporation. The study demonstrated that these exosomes successfully reduced tumor growth, induced apoptosis, and prolonged survival (*p* < 0.001) in a PCa model.^[^ ^86^ ^]^ In another study, the authors have confirmed that both miR‐34a‐5 and si‐c‐MYC can reduce the growth of CRC and prevent EMT through exosomal delivery. Exosomes collected from MSCs that had been transfected with either miR‐34a‐5p mimic or Si‐c‐MYC significantly reduced tumor growth and metastasis both in vitro and in vivo.^[^ ^87^ ^]^

Surface modification of exosomes can enhance their target specificity, thereby improving the efficacy of tumor growth inhibition and proliferation suppression by optimizing chemotherapeutic agent delivery. In a recent study, EGFR‐targeting GE11 peptides were conjugated to the surface of oxaliplatin‐loaded exosomes to inhibit progression of CRC. The conjugation of GE11 peptides to oxaliplatin‐loaded exosomes significantly enhanced their uptake by EGFR‐expressing cancer cells, leading to increased apoptosis and reduce cancer cell viability in a CRC xenograft model.^[^ ^88^ ^]^ In another study, the surface of PC cell derived vesicles loaded with paclitaxel (PTC) was modified with αvβ3‐targeting RGD peptides and magnetic nanoparticles to enhance target specificity. The engineered EVs significantly reduced tumor size in a PANC‐1 xenograft model, demonstrating enhanced tumor targeting and penetration due to affinity for αvβ3 integrin, with the therapeutic effect attributed to the homing properties of the exosomes and mediated by integrin β3 (Figure 4A–G).^[^ ^89^ ^]^

Aptamers, frequently used in exosome engineering, are short nucleotide sequences that specifically bind to target molecules with high affinity. In a study by Hosseini et al., HEK293 cell derived exosomes were loaded with DOX, followed by surface modification with AS1411 (anti‐nucleolin) aptamers. These exosomes exhibited strong binding affinity and high cellular uptake in nucleolin‐overexpressing cancer cells. As a result, the aptamer‐functionalized exosomes effectively suppressed tumor growth in a colon cancer mouse model through targeted drug delivery and long‐term retention at the tumor site. Similarly, Bagheri et al. attached MUC1‐targeting aptamers on MSC‐derived exosomes, which were loaded with DOX using electroporation. These exosomes demonstrated high specificity to MUC1‐positive cancer cells in vitro and significantly suppressed tumor growth in vivo.^[^ ^90^ ^]^

Loading of nucleic acids and surface modification in exosomes can be done simultaneously to selectively inhibit the tumor growth. Exosomes derived from iRGD‐modified cord blood MSCs were used to target NRP‐1, which is overexpressed in CRC. These exosomes were then loaded with anti‐miRNA‐221 oligonucleotides (AMOs) to inhibit the progression of CRC. In a xenograft model, iRGD‐modified, AMO‐loaded exosomes successfully accumulated at the tumor site, significantly reducing tumor progression.^[^ ^91^ ^]^ Xiang and colleagues used 293T cell‐derived exosomes to deliver miR‐26a, which is known to induce apoptosis in HCC. The 293T cells were initially transfected with Apo‐CD63 plasmid using Lipofectamine. The exosomes derived from these cells were found to overexpress Apo‐A1, a well‐established target of scavenger receptor class B type 1 (SR‐B1), which is highly expressed in HCC. Following the exosome isolation, miR‐26a was encapsulated into the exosomes via electroporation, and the exosomal delivery of these miRNAs upregulated miR‐26a expression and inhibited cell growth in HepG2 cells. Specifically, the delivery of these engineered exosomes led to the downregulation of Cyclin D2, Cyclin E2, and CDK6 levels, resulting in cell cycle arrest and the inhibition of cell proliferation, as well as metastasis in HCC.^[^ ^92^ ^]^

Superparamagnetic nanoparticles are also frequently used in exosome engineering. In a study conducted by Master et al., exosomes collected from A33‐positivie LIM1215 cells were loaded with DOX using electroporation. These exosomes were incubated with superparamagnetic iron oxide nanoparticles (US) functionalized with anti‐A33 antibodies. This exosome complex exhibited strong binding affinity, enhanced uptake, and antiproliferative effects in LIM1215 cells, effectively targeted tumors, inhibited growth, prolonged survival, and reduced cardiotoxicity in vivo.^[^ ^93^ ^]^

#### 3.2.3 Limitations and Aspects for Exosome‐Based Growth and Proliferation Inhibition

While exosomes present a promising platform for the delivery of therapeutic agents to inhibit tumor growth and metastasis, several limitations need to be addressed to maximize their therapeutic efficacy in clinical applications. One major challenge is the scalability of exosome production. Currently, isolating and purifying exosomes in large quantities remains labor‐intensive and costly, hindering the consistent production of therapeutically viable batches. Additionally, the heterogeneity of exosomes, influenced by their cell of origin, may lead to variability in therapeutic efficacy and targeting specificity, which raises concerns about standardization and quality control in exosome‐based therapies.

Moreover, the limited understanding of exosome biodistribution and clearance mechanisms in vivo presents another obstacle. While exosomes are generally considered to have low immunogenicity, their clearance by the liver and spleen can reduce their therapeutic efficacy in targeting tumors. Future research should focus on enhancing the stability and circulation time of exosomes, potentially through surface modifications or encapsulation techniques, to improve their retention at the tumor site. Advances in surface engineering, such as the incorporation of targeting ligands and peptides, have shown potential, but further studies are needed to optimize these strategies for different cancer types and patient populations. Addressing these challenges will be crucial for translating exosome‐based therapies from experimental models to clinical use in inhibiting cancer growth and metastasis.

### 3.3 Exosomes for Reversing Drug Resistance

#### 3.3.1 Exosomes as Therapeutic Agents for Reversing Drug Resistance

Drug resistance in tumor cells can be classified as intrinsic or acquired. Intrinsic resistance refers to the pre‐existing, inherent inability of cancer cells to respond to therapeutic agents at the outset of treatment. In contrast, acquired resistance develops over time as cancer cells, initially sensitive to treatment, adapt and become resistant following prolonged or repeated drug exposure. When a tumor acquires drug resistance, the therapeutic efficacy of these agents is significantly diminished. Even if the bulk of the tumor is eradicated, a small population of drug‐resistant cancer cells can persist, potentially leading to recurrence and reducing the effectiveness of subsequent chemotherapy regimens. The emergence of drug‐resistant cancer cells represents a major barrier to successful cancer therapy. Various factors are known to contribute to drug resistance, including irregular gene expression, overexpression of transporters like P‐glycoprotein, and metabolic detoxification.^[^ ^97^ ^]^ These factors can pose challenges for cancer treatment.

Exosomes have been explored as potential agents for counteract drug resistance, potentially enhancing antitumor activity. Exosomes can carry and deliver bioactive molecules such as small RNAs, proteins, and chemotherapeutic drugs, exosomes can help modulate the tumor microenvironment and counteract drug resistance mechanisms. One of the key advantages of exosomes is their ability to transfer therapeutic cargo to drug‐resistant cancer cells by bypassing traditional drug efflux mechanisms, such as those mediated by P‐glycoprotein. By encapsulating chemotherapeutic agents or RNA‐based therapeutics, exosomes can deliver these molecules directly into cancer cells, potentially reducing the impact of metabolic detoxification pathways that typically diminish drug efficacy.

Furthermore, exosomes can be engineered to target specific molecules that are over‐expressed in drug‐resistant cells, enhancing their ability to overcome both intrinsic and acquired resistance. These approaches not only resensitize resistant cancer cells to standard therapies but can also inhibit the survival and proliferation of resistant cell populations. As such, exosome‐based strategies offer a novel approach to address one of the most significant challenges in cancer therapy—overcoming drug resistance and improving patient outcomes in resistant tumor types.

#### 3.3.2 Therapeutic Potential of Exosomes for Reversing Drug Resistance

MiR‐214, which is often overexpressed in tumors, is known to be associated with drug resistance to cisplatin (DDP). In a study conducted by Wang et al., HEK293T cells transfected with miR‐214 inhibitors have been utilized to enhance the efficacy of cisplatin (**Table** **6**). Exosomes carrying anti‐miR‐214 were shown to reverse cisplatin resistance in GC by sensitizing cells to the drug in vitro, inhibiting tumor growth, and enhancing treatment efficacy in vivo.^[^ ^98^ ^]^ Suppressing the c‐MET expression is another way to reduce drug resistance upon DDP treatment. Zhang et al. collected exosomes from HEK293T cells transfected with si‐c‐MET and discovered that delivering si‐c‐MET through these exosomes can substantially overcome cisplatin resistance both in vitro and in vivo.

**Table 6:** Summary of the exosome‐based therapeutics for reversing the drug resistance in gastrointestinal (GI) cancer.

Drugs	Exosomes		Encapsulation/loading		Surface modification		Therapeutic outcomes	Refs.
	Source	Isolation	Cargoes	Method	Molecules	Method		
Cisplatin	HEK293T	Ultracentrifugation (UC)	Anti‐miR‐214	Transfection of parental cells using Lipofectamine	N/A	N/A	Anti‐miR‐214 carrying exosomes reverses cisplatin resistance in GC by downregulating miR‐214, reducing cell viability, and promoting apoptosis	[98]
Cisplatin	HEK293T	UC	si‐c‐Met	Transfection of parental cells using Lipofectamine	N/A	N/A	Exosomal delivery of si‐c‐Met reverses cisplatin resistance in GC by reducing c‐Met expression, and promoting apoptosis	[107]
5‐Fluorouracil (5‐FU)	Cancer cell lines	UC	sh‐circ0000338	Transfection of parental cells using Lipofectamine	N/A	N/A	Exosomal circ_0000338 enhances 5‐FU resistance in CRC by suppressing miR‐217 and miR‐485‐3p, whereas exosomal delivery of sh‐circ0000338 reverse the resistance	[100]
5‐FU	HEK293T	UC	miR21 inhibitor 5‐FU	Electroporation	HER2‐binding affibody	Transfection of parental cells using lentivirus vector	Co‐delivery of miR‐21i and 5‐FU via engineered exosomes sensitized 5‐FU‐resistant colon cancer cells, reducing tumor proliferation and promoting apoptosis	[82]
Oxaliplatin	FHC cells	UC	miR‐1915‐3p	Transfection of parental cells with lentiviral vector	N/A	N/A	Exosomal delivery of miR‐1915‐3p restored oxaliplatin sensitivity in resistant CRC cells by reducing epithelial‐to‐mesenchymal transition (EMT) markers and oncogene expression (PFKFB3, USP2), promoting apoptosis and reducing migration.	[102]
Oxaliplatin	FHC cells	UC	circ‐FBXW7	Transfection of parental cells using Lipofectamine	N/A	N/A	Exosomal delivery of circ‐FBXW7 restored oxaliplatin sensitivity in resistant CRC cells by sponging miR‐18b‐5p, promoting apoptosis, inhibiting EMT, and reducing drug efflux	[108]
Oxaliplatin	HEK293T	UC	PGM5‐AS1 Oxaliplatin	Transfection of parental cells with lentiviral vector (PGM5‐AS1) Electorporation (Oxaliplatin)	N/A	N/A	Exosomal delivery of PGM5‐AS1 and oxaliplatin restored oxaliplatin sensitivity in resistant CRC cells by promoting apoptosis, downregulating progestagen associated endometrial protein (PAEP), and upregulating NME1.	[103]
Gemcitabine	Melanoma cells	UC	Survivin‐T34A	Transfection of parental cells using tetracycline‐regulated system	N/A	N/A	Exosomal delivery of Survivin‐T34A increased apoptosis and enhanced gemcitabine sensitivity in pancreatic cancer (PCa) cells.	[104]
Gemcitabine Paclitaxel	MSCs	UC	Gemcitabine Paclitaxel	Electroporation	N/A	N/A	Exosomal delivery of GEMP and PTX enhanced gemcitabine sensitivity in pancreatic ductal adenocarcinoma (PDAC) by improving tumor penetration and reducing chemoresistance.	[105]
Doxorubicin (DOX)	MSCs	UC	MiR‐199a‐3p	Transfection of parental cells with lentiviral vector	N/A	N/A	Exosomal delivery of miR‐199a‐3p sensitized HCC cells to DOX by inhibiting the mTOR pathway and enhancing apoptosis.	​[106]

**Figure 5:**
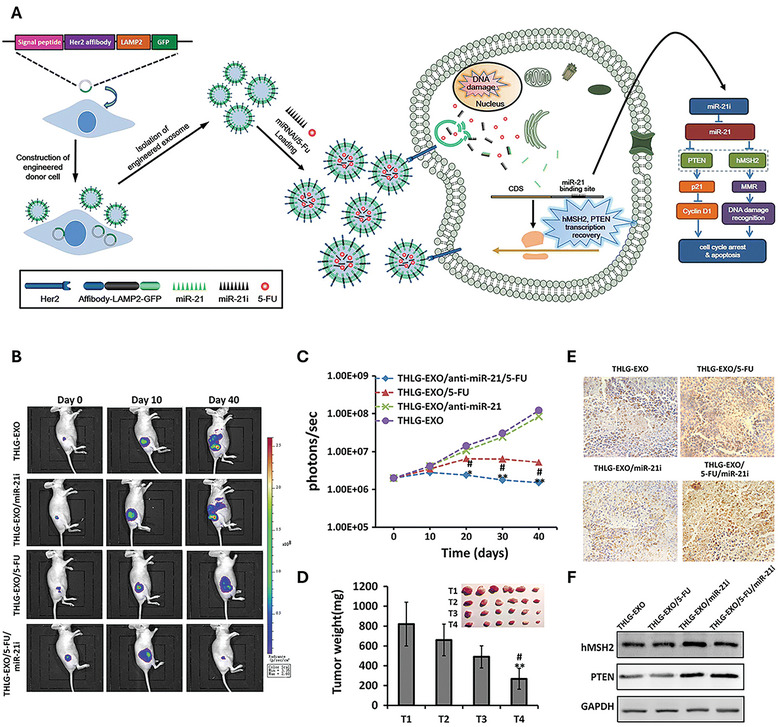
Engineered extracellular vesicles (EVs) for reversing the drug resistance: A) Engineered exosomes for 5‐FU and miR‐21i delivery to enhance the therapeutic efficacy. B) Representative bioluminescent images demonstrating tumor growth in nude mice treated with THLG‐EXO, THLG‐EXO/miR‐21i, THLG‐EXO/5‐FU, or THLG‐EXO/5‐FU/miR‐21i at various time points. Signals are adjusted to a consistent color scale. C) The mean bioluminescence intensity (BLI) for each mouse/group, quantifying the relative bioluminescent intensity at the tumor site. D) Tumor weights from different treatment groups (THLG‐EXO, THLG‐EXO/miR‐21i, THLG‐EXO/5‐FU, THLG‐EXO/5‐FU/miR‐21i) presented as mean ± SD. E) TUNEL staining of tumor tissues treated with THLG‐EXO, THLG‐EXO/miR‐21i, THLG‐EXO/5‐FU, or THLG‐EXO/5‐FU/miR‐21i, indicating apoptosis levels. F) Western blot analysis demonstrating the expression of hMSH2 and PTEN in tumor tissues 24 hours post‐administration. Data are expressed as mean ± SD (*n* = 6); ^*^ *p* < 0.05, ^**^ *p* < 0.01 versus THLG‐EXO or THLG‐EXO/miR‐21i groups, and ^#^ *p* < 0.05 versus THLG‐EXO/5‐FU group. Reproduced with permission.^[^ ^82^ ^]^ Copyright 2020, Springer Nature.

5‐Fluorouracil (5‐FU) is a widely used chemotherapeutic agent for the treatment of various malignancies. However, its therapeutic efficacy is often compromised by the development of resistance in tumor cells, posing a significant challenge to successful treatment outcomes.^[^ ^99^ ^]^ A recent study revealed that elevated expression of exosomal circ_0000338 is associated with 5‐FU resistance, as patients with 5‐FU‐resistant CRC exhibited higher levels of exosomal circ_0000338. The study demonstrated that the exosomal transfer of circ_0000338 inhibits miR‐217 and miR‐485‐3p, which ultimately increases resistance to 5‐FU. In this context, downregulation of circ_0000338 through the exosomal delivery of sh‐circ_0000338 successfully reversed 5‐FU resistance.^[^ ^100^ ^]^ MiR‐21 is also known to promote 5‐FU resistance in CRC by downregulating the expression of human DNA MutS homolog 2 (hMSH2).^[^ ^101^ ^]^ In a study conducted by Liang et al., exosomes were engineered to co‐deliver 5‐FU and a miR‐21 inhibitor to 5‐FU‐resistant CRC cells. Specifically, the exosomes were modified by incorporating a Her2‐LAMP2 fusion protein, enabling targeted delivery to HER2‐expressing tumor cells, and loaded with 5‐FU and miR‐21i using electroporation. The co‐delivery of miR‐21i and 5‐FU using the engineered exosomes significantly enhanced the sensitivity of 5‐FU‐resistant cancer cells to chemotherapy. This approach effectively reduced tumor cell proliferation, induced apoptosis, and inhibited tumor growth in both in vitro and in vivo models, demonstrating a strong potential to reverse drug resistance (**Figure** **5**).^[^ ^82^ ^]^

Oxaliplatin is a platinum‐based chemotherapeutic drug commonly used to treat CRC by inducing DNA crosslinks and inhibiting DNA replication. In a study conducted by Xiao et al., the exosomes isolated from miR‐1915‐3p‐transduced FHC cells were utilized to enhance the efficacy of oxaliplatin. The delivery of miR‐1915‐3p via exosomes significantly restored oxaliplatin sensitivity in CRC cells by suppressing EMT‐promoting oncogenes (PFKFB3 and USP2), reducing cell migration, invasion, and enhancing apoptosis both in vitro and in vivo.^[^ ^102^ ^]^ Similarly, exosomal circ‐FBXW7 was investigated for its ability to reverse oxaliplatin resistance in CRC. The researchers found that circ‐FBXW7 levels were lower in oxaliplatin‐resistant CRC patients. Exosomes containing circ‐FBXW7, derived from circ‐FBXW7‐transfected FHC cells, were able to transfer this RNA to resistant CRC cells, significantly enhancing their sensitivity to oxaliplatin. The mechanism involved sponging miR‐18b‐5p, a microRNA overexpressed in resistant CRC cells, which in turn restored apoptotic pathways, inhibited epithelial–mesenchymal transition, and reduced drug efflux. Both in vitro and in vivo, exosomal circ‐FBXW7 improved oxaliplatin efficacy, offering a potential therapeutic approach for overcoming chemoresistance in CRC.^[^ ^103^ ^]^ In another study, Hui et al. explored the role of engineered exosomes for the co‐delivery of PGM5‐AS1 and oxaliplatin in reversing oxaliplatin resistance in CRC. PGM5‐AS1, a long non‐coding RNA (lncRNA), was found to be downregulated in oxaliplatin‐resistant CRC tissues and cells. The researchers engineered exosomes derived from 293T cells to carry both PGM5‐AS1 and oxaliplatin via electroporation. These exosomes were shown to increase the expression of PGM5‐AS1 in CRC cells, reduce oxaliplatin resistance, and inhibit cell proliferation and metastasis. The combination of PGM5‐AS1 and oxaliplatin delivered through exosomes effectively restored sensitivity to oxaliplatin in vitro and in vivo by promoting apoptosis and downregulating progestagen associated endometrial protein (PAEP), while upregulating NME1, both of which were identified as key modulators of the malignant phenotype in CRC.^[^ ^103^ ^]^

Gemcitabine is a nucleoside analog commonly used to treat PDAC by inhibiting DNA synthesis and promoting apoptosis. However, its efficacy is limited by various chemoresistance mechanisms in PDAC cells. In a study by Aspe et al., exosomes isolated from melanoma cells overexpressing the Survivin‐T34A mutant were used to sensitize PCa cells to gemcitabine. Survivin, an inhibitor of apoptosis protein, is known to contribute to gemcitabine resistance. Exosomes containing the Survivin‐T34A mutant delivered to PDAC cells in combination with gemcitabine significantly increased apoptotic cell death compared to gemcitabine alone. The combination treatment disrupted Survivin‐mediated resistance pathways, enhancing the chemosensitivity of PDAC cells in vitro, suggesting a potential therapeutic strategy.^[^ ^104^ ^]^ In another study, exosomes derived from bone marrow MSCs (BM‐MSCs) were utilized to enhance the penetration and efficacy of chemodrugs. By co‐loading gemcitabine monophosphate and paclitaxel into BM‐MSC‐derived exosomes via electroporation, the researchers significantly overcame the chemoresistance of PDAC cells. These exosomes exhibited superior tumor‐homing abilities, penetrated the dense extracellular matrix, and accumulated at the tumor site. The exosomal delivery of GEMP and PTX improved apoptosis rates and reduced tumor growth in vitro and in vivo, offering a promising strategy for overcoming gemcitabine resistance in PDAC.^[^ ^105^ ^]^

DOX is a widely used chemotherapeutic agent in the treatment of tumors, including HCC. However, its efficacy is often reduced due to the development of chemoresistance in HCC cells. In a study by Lou et al., exosomes derived from adipose tissue MSCs (AMSCs) were engineered to carry miR‐199a‐3p, a microRNA known to be downregulated in HCC. MiR‐199a‐3p was loaded into AMSCs through lentiviral transfection and the exosome obtained from these cells effectively delivered miR‐199a‐3p to HCC cells, targeting the mTOR pathway, which plays a key role in chemoresistance. The combination of AMSC‐Exo‐199a and DOX significantly inhibited cell proliferation and enhanced apoptosis in vitro, and the co‐treatment also markedly reduced tumor growth in vivo. This suggests that AMSC‐Exo‐199a‐mediated miR‐199a‐3p delivery can overcome DOX resistance by modulating the mTOR pathway​^[^ ^106^ ^]^

#### 3.3.3 Limitations and Aspects for Reducing Drug Resistance Using Exosomes

While exosome‐based systems show significant potential in overcoming drug resistance in cancer therapy, several limitations must be addressed to realize their full therapeutic potential. One of the major challenges in this approach is the heterogeneity of exosome populations. Exosomes derived from different cell types or even from the same cells under varying conditions may carry different cargoes, leading to inconsistent therapeutic outcomes. This variability complicates their application in reducing drug resistance, as the specific exosomal contents responsible for reversing resistance, such as miRNAs, proteins, or small molecules, may not be uniformly present across all exosomes. Consistency in exosome cargo is critical for effective and reproducible results in drug‐resistant cancer treatments.

Efficient loading of therapeutic cargoes into exosomes remains another technical hurdle. Methods like electroporation, co‐incubation, or transfection are commonly used to load drugs, miRNAs, or other therapeutic agents into exosomes. However, these methods often result in low encapsulation efficiency and potential damage to exosomal membranes, reducing the effectiveness of drug delivery. Optimizing loading techniques to enhance the stability and therapeutic potency of exosome‐encapsulated agents is an area of ongoing research.

Another significant limitation is the lack of specific targeting. While exosomes have some inherent tumor‐targeting abilities due to their ability to mimic natural cell communication, their distribution in the body is often non‐specific. This results in a portion of exosomes being taken up by healthy tissues or rapidly cleared by the liver and spleen. Enhancing the targeting specificity of exosomes to drug‐resistant cancer cells is crucial. Surface modifications using peptides, antibodies, or aptamers are potential strategies to improve exosome homing to resistant tumor cells, but these approaches need further optimization to ensure they are effective across different cancer types and stages.

The biological complexity of drug resistance mechanisms also presents a challenge. Drug resistance in cancer cells often involves multiple pathways and cellular changes, including alterations in drug efflux pumps, apoptotic pathways, and survival signaling networks. Exosome‐based systems must be able to deliver their cargoes in a way that effectively modulates these complex, multifaceted resistance mechanisms. Achieving the right balance of therapeutic agents in exosomes to counteract diverse resistance pathways, while minimizing off‐target effects, remains an ongoing challenge. Addressing these limitations is crucial for developing exosome‐based therapies to reverse drug resistance. Improvements in exosome production, targeting specificity, and loading efficiency will facilitate for more effective treatments that can restore drug sensitivity in resistant cancer cells, offering new strategy to efficiently fight against GI cancer.

### 3.4 Exosomes for Cancer Immunotherapy

#### 3.4.1 Exosomes as Therapeutic Agents for Cancer Immunotherapy

Cancer immunotherapy has transformed cancer treatment by engaging the immune system to recognize and eliminate cancer cells. Unlike chemotherapy and radiation, which directly target tumor cells but often damage healthy tissues, immunotherapy harnesses the immune system's specificity to selectively attack malignant cells.^[^ ^109^ ^]^ Immune checkpoint inhibitors, CAR T‐cell therapies, and cancer vaccines have demonstrated significant efficacy in multiple cancer types, highlighting immunotherapy as a promising approach for durable cancer remission and fewer side effects. However, immunotherapies face limitations, including tumor immune evasion, off‐target immune responses, and poor penetration into certain tumor microenvironments. These challenges highlight the need for more precise approaches to enhance immune activation against cancer cells.^[^ ^110^ ^]^

Exosomes have emerged as promising agents for cancer immunotherapy due to their role in intercellular communication and their ability to transport bioactive molecules. Derived from different cell types, exosomes encapsulate proteins, lipids, and nucleic acids that reflect their cell of origin, enabling them to modulate immune responses in both physiological and therapeutic settings.^[^ ^111^ ^]^ Their nanoscale size allows exosomes to efficiently penetrate biological barriers, providing a low‐immunogenic, customizable platform for delivering antigens and immune‐modulatory molecules to immune cells and tumor sites.

Specifically, exosomes derived from DCs and loaded with tumor antigens can initiate a tumor‐specific immune response by presenting these antigens to T cells, thereby activating the adaptive immune system to recognize and target cancer cells. Additionally, tumor‐derived exosomes can be engineered to carry specific antigens or immune‐stimulating agents that prompt an anti‐tumor immune response. Beyond antigen presentation, exosomes engineered to inhibit immune checkpoints or deliver checkpoint blockade antibodies (e.g., anti‐PD‐1 or anti‐PD‐L1) can counteract immunosuppressive signals in the tumor microenvironment, enhancing anti‐tumor immunity.^[^ ^112^ ^]^ Through these versatile functions, exosomes present a novel and promising strategy to overcome the current challenges of immunotherapy, offering a more effective and personalized approach to cancer treatment.

#### 3.4.2 Therapeutic Potential of Exosomes for Cancer Immunotherapy

**Figure 6:**
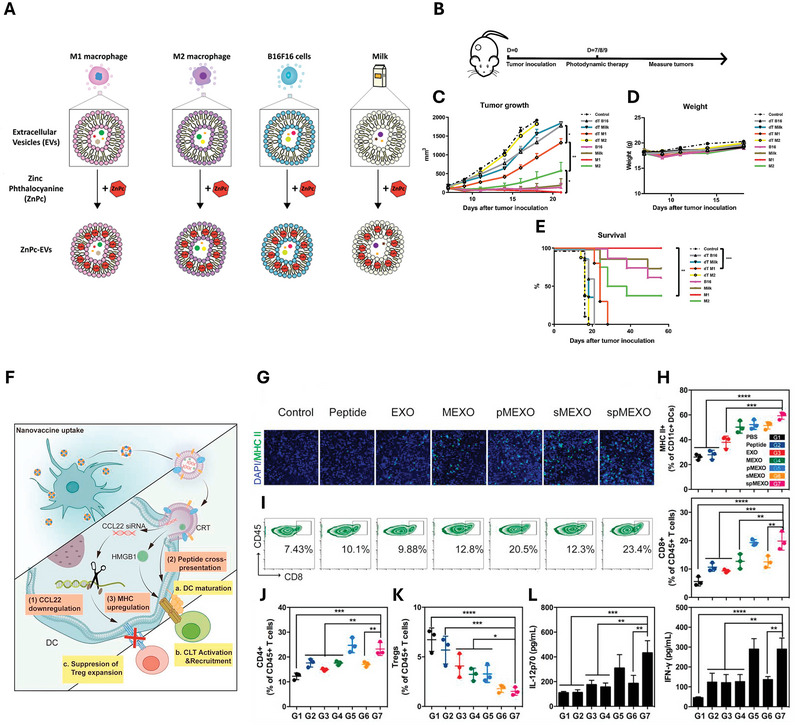
Engineered extracellular vesicles (EVs) for modulating immune responses. A) EVs containing the photosensitizer Zinc Phthalocyanine (ZnPc) for enhanced antitumor efficacy via EV‐mediated photodynamic therapy (PDT). B) In vivo study design. C,D) Tumor growth and total among different treatment groups. E) Survival analysis among different treatment groups. (^*^ *p* < 0.05; ^**^ *p* < 0.01; ^***^ *p* < 0.0001; mean ± SEM, *n* ≥ 7). Reproduced with permission.^[^ ^118^ ^]^ Copyright 2022, Springer Nature. F) The immunostimulatory effect of nanovaccine spMEXO. G) Immunofluorescence imaging of MHC II+ dendritic cells (DCs) upon treatment with engineered EVs (green: MHC II; blue: DAPI; magnification 200×). H) Flow cytometry analysis for the quantification of MHC II‐expressing DCs upon treatment with the engineered EVs. I) Flow cytometry analysis of CD8+ T cells, J) CD4+ T helper cells K) regulatory T cells (Tregs) after co‐incubation with pretreated DCs. L) Expression levels of IL‐12p70 and IFN‐γ in cocultured medium from DCs and T cells upon treatment with the engineered EVs (mean ± SD, one‐way ANOVA and Tukey multiple comparisons tests, ^*^ *p* < 0.05, ^**^ *p* < 0.01, ^***^ *p* < 0.001, ^****^ *p* < 0.0001). Reproduced with permission.^[^ ^114^ ^]^ Copyright 2022, Elsevier.

Tumor‐derived exosomes (TDEs) can be utilized for cancer immunotherapy by delivering tumor‐associated antigens or immune‐stimulating molecules to immune cells, thereby enhancing anti‐tumor responses (**Table** **7**). These exosomes can be further engineered to deliver specific miRNAs, proteins, or antigens that modulate the immune environment by promoting CD8+ T cell activation and reducing immunosuppressive pathways. Additionally, TDEs can be designed to inhibit immune checkpoint molecules (e.g., PD‐L1) in the tumor microenvironment, reducing immune evasion and enhancing the immune system's ability to target and destroy cancer cells. In a study conducted by Hosseini et al., miR‐34a was loaded into TDE obtained from CT‐26 CRC cells using a modified CaCl₂ transfection method, which involved mixing the miR‐34a mimic with TDE, followed by a brief heat shock and RNase treatment to remove unincorporated miRNAs​. These miR‐34a‐enriched exosomes stimulated CD8+ T cell activation by reducing the expression of immune evasion markers, such as PD‐L1, in the tumor microenvironment, leading to an increased CD8+/CD4+ T cell ratio and enhancing anti‐tumor immune responses in vivo.^[^ ^113^ ^]^ In another study, nanovaccine was developed using exosomes derived from immunogenically dying PANC‐02 PCa cells, engineered with MART‐1 peptides on the surface and loaded with CCL22 siRNA through electroporation. The MART‐1 peptides enhanced T‐cell activation, while the CCL22 siRNA disrupted the immunosuppressive CCR4/CCL22 axis, reducing Treg recruitment. In vivo study revealed that these engineered exosomes (spMEXO) significantly delayed tumor growth and improved immune response, especially when combined with chemotherapy, highlighting their potential in PCa immunotherapy (**Figure** **6**F–L).^[^ ^114^ ^]^

**Table 7:** Summary of the exosome‐based therapeutics for immunotherapy in gastrointestinal (GI) cancer.

Exosomes		Encapsulation/loading		Surface modification		Therapeutic outcomes	Refs.
Source	Isolation	Cargoes	Method	Molecules	Method		
Murine colon cancer cell line CT‐26	Exocib Kit	Mir‐34a	Loaded onto exosomes using CaC1_2_ solution	N/A	N/A	Significant reduction in tumor size, increased CD8+ T cell polarization, reduced oncogenic gene expression, and enhanced anti‐tumor immune response in colorectal cancer (CRC)	[113]
Immunogenically dying PANC‐02 cells	Ultracentrifugation (UC) (iodixanol‐sucrose gradient)	CCL22 siRNA	Electroporation	MART‐1 peptide (MHC class I targeting peptide)	MART‐1 peptide was linked to C05 peptides which binds to CD63 on exosomes	Enhanced antigen presentation, T‐cell activation, inhibition of CCR4/CCL22 axis to suppress Tregs, increased therapeutic efficacy in pancreatic ductal adenocarcinoma (PDAC) when combined with chemotherapy	[114]
Mature DCs	UC	Alpha‐fetoprotein (AFP) gene	Transfection of parental cells using recombinant adeno‐associated viral vector (rAAV)‐carrying alpha‐fetoprotein (*AFP*) gene (rAAV/AFP)	N/A	N/A	AFP gene‐loaded dendritic cell‐derived exosome (DEX) stimulate naïve T cells to become antigen specific cytotoxic T lymphocytes (CTLs), enhancing antitumor responses against hepatocellular carcinoma (HCC)	[120]
Dendritic cell line DC2.4	UC	Neoantigen peptides (M27, M30, Adpgk)	Electroporation	N/A	N/A	Elicited strong T‐cell and B‐cell immune responses, delayed tumor growth, increased survival, inhibited metastasis, and induced long‐term memory in melanoma and CRC models	[115]
Human NK cells	UC	N/A	N/A	N/A	N/A(	Induced apoptosis and inhibited proliferation in HCC cells via intrinsic and extrinsic apoptotic pathways; targeted tumor tissue and inhibited tumor growth in both subcutaneous and orthotopic HCC models	[116]
M1‐like macrophage	UC	Zinc phthalocyanine (ZnPc)	Incubation	N/A	N/A	Enhanced photodynamic therapy efficacy, induced immunogenic cell death, dendritic cell maturation, and immunological memory; effectively delayed tumor growth in colon cancer models	​[118]
CAR‐engineered primary T cells	UC	N/A	N/A	4D5‐5 scFv (HER2‐targeting)	T cells were transduced by lentiviral vector	Exhibited specific cytotoxic activity against tumor cells without programmed death 1 (PD‐1) expression, preventing immune evasion by PD‐L1+ tumors, and induced potent antitumor effects with lower toxicity than CAR‐T cells	[119]

Dendritic cell‐derived exosomes (DEXs) play a significant role in cancer immunotherapy by acting as natural carriers of immune‐activating molecules. These exosomes contain MHC‐peptide complexes and co‐stimulatory molecules that can directly present tumor antigens to T cells, leading to the activation and proliferation of cytotoxic T lymphocytes (CTLs). Additionally, DEX can be engineered to carry specific tumor antigens or immune‐modulating molecules, further enhancing their capacity to stimulate anti‐tumor immunity. By promoting a robust CTL response and improving antigen presentation, DEX‐based therapies offer a promising, targeted approach for enhancing immune surveillance and reducing tumor growth in cancer patients. In a study conducted by Zhou et al, the researchers utilized exosomes derived from DCs transfected with a recombinant adeno‐associated virus carrying the alpha‐fetoprotein (AFP) gene. These engineered DEX carried MHC‐I, MHC‐II, and co‐stimulatory molecules to effectively present antigens to T cells, thereby stimulating naive T cells to become CTLs. Therapeutically, these DEX successfully induced AFP‐specific CTL responses, significantly increasing anti‐tumor immune activity against HCC by enhancing T cell proliferation and IFN‐γ secretion, showing potential for targeted cancer immunotherapy​. In another study, exosomes were derived from DCs (DC2.4 cell line) and loaded with the Adpgk neoantigen peptide specific to MC‐38 CRC cells using electroporation. This exosome‐based vaccine, termed Exo‐Adpgk, was designed to enhance immune response by promoting CTL and T‐helper cell infiltration into tumors, significantly inhibiting tumor growth in the MC‐38 model. The therapeutic effect demonstrated that Exo‐Adpgk increased CD8+ T cell activity and provided a more potent antitumor response compared to liposome‐loaded neoantigen formulations.^[^ ^115^ ^]^

NK cell‐derived exosomes also have shown potential to be utilized for cancer immunotherapy in GI cancers due to their inherent cytotoxic components, such as perforin, granzymes, and TRAIL, which can directly induce apoptosis in cancer cells. These exosomes can also be engineered or loaded with additional therapeutic agents to enhance their tumor‐targeting capabilities and efficacy, thereby boosting their immunotherapeutic effects. Additionally, NK exosomes contribute to immune modulation in the tumor microenvironment, enhancing anti‐tumor immune responses and overcoming immune evasion mechanisms typically present in GI cancers. In a study conducted by Kim et al., NK cell‐derived exosomes (NK‐exo) were obtained from NK‐92 cells and characterized by their high content of cytotoxic proteins, such as perforin, granzyme B, TRAIL, and FasL. These NK‐exo were selectively taken up by HCC cells, where they triggered apoptosis by activating both intrinsic and extrinsic pathways, involving caspase activation and the release of mitochondrial cytochrome c. Therapeutically, NK‐exo significantly inhibited tumor growth and enhanced survival in both orthotopic and subcutaneous HCC mouse models, demonstrating strong tumor‐targeting and anti‐tumor efficacy through apoptotic mechanisms without affecting normal liver cells​.^[^ ^116^ ^]^ Although research has primarily focused on other cancers such as breast, ovarian, and melanoma, strategies involving the loading of chemotherapeutic drugs (e.g., cisplatin or paclitaxel) or nucleic acids (e.g., miR‐31/193b) onto NK‐exo have been shown to enhance therapeutic effects.^[^ ^117^ ^]^ These approaches hold potential for GI tumors as well, further activating the immune system and inhibiting tumor growth.

Exosomes obtained from other types of immune cells are also being investigated as a drug delivery vehicles for modulating immune systems. Veld et al. used exosomes derived from M1‐polarized macrophages, loaded with the photosensitizer Zinc Phthalocyanine (ZnPc) for photodynamic therapy (PDT) in colon cancer. ZnPc was incorporated into the exosomes through incubation, enabling targeted PDT that induced immunogenic cell death (ICD) in cancer cells by exposing damage‐associated molecular patterns (DAMPs), which enhanced DC maturation. Therapeutically, M1‐ZnPc exosomes showed selective toxicity to cancer cells, delayed tumor growth, and promoted immune memory in a murine colon cancer model, highlighting their potential in inducing long‐term anti‐tumor immunity ​(Figure 6A–E).^[^ ^118^ ^]^ Exosomes can be also obtained from chimeric antigen receptor (CAR) T cells. In a study conducted by Fu et al., CAR‐T cells were engineered to target HER2‐positive tumor cells, expressing high levels of cytotoxic proteins, including granzyme B and perforin, on their surface. The CAR exosomes demonstrated specific binding to HER2‐expressing cancer cells, inducing apoptosis and effectively inhibiting tumor growth in vivo with a lower risk of toxicity compared to CAR‐T cells. Additionally, CAR exosomes showed resilience against PD‐L1‐mediated immunosuppression, suggesting their advantage in solid tumor environments where immune evasion is prevalent. This research highlights the strong therapeutic potential of CAR exosomes for targeted cancer immunotherapy.^[^ ^119^ ^]^

#### 3.4.3 Limitations and Aspects for Exosome‐based Cancer Immunotherapy

Exosome‐based cancer immunotherapy offers significant promise, yet several key challenges must be addressed to optimize clinical outcomes. A primary limitation is the potential for immune clearance and off‐target effects due to the complex biodistribution patterns of exosomes. Unlike direct immune cell‐based therapies, exosomes are rapidly taken up by organs like the liver and spleen, which reduces their concentration at the tumor site. This limits the therapeutic efficacy of exosome‐based approaches in solid tumors, particularly those in GI cancers, where deep tissue penetration is necessary.

Additionally, the scalability of exosome production presents a logistical challenge. Current methods allow only limited quantities of immune cells to be obtained from patients, making the generation of large numbers of exosomes difficult. This limitation is compounded by the costly and labor‐intensive processes required for exosome isolation and purification, which hinder the ability to produce therapeutic doses at a large scale. To make exosome‐based immunotherapies feasible for widespread clinical applications, there is a pressing need to develop efficient, scalable, and cost‐effective production methods.

Furthermore, heterogeneity in exosome content and characteristics, depending on the source cells, may impact the reliability and consistency of therapeutic outcomes. Each type of immune cell‐derived exosome, whether from NK cells, DCs, or others, carries distinct surface markers and bioactive molecules, leading to variable immune activation. To overcome this, enhanced engineering techniques to load specific therapeutic agents, alongside targeted surface modifications, are essential. Achieving standardized exosome formulations will improve targeting and therapeutic efficiency, advancing the potential of exosome‐based immunotherapy for GI cancers.

## 4 Discussion

Exosome‐based strategies have rapidly emerged as a transformative approach in the diagnosis of GI cancers. Exosomes, tiny vesicles carrying biomolecules such as miRNAs, lncRNAs, circRNAs, and proteins, serve as stable and specific biomarkers detectable in various body fluids. The studies reviewed significant advancements in identifying exosomal biomarkers for early GI cancer detection, cancer type differentiation, and prognosis prediction. For instance, exosomal miRNAs such as miR‐4772‐3p and miR‐6803‐5p exhibit high specificity and sensitivity in diagnosing colon and CRCs, respectively. Similarly, exosomal lncRNAs and circRNAs have demonstrated potential in not only diagnosing but also providing insights into the tumor's origin and progression. Despite challenges such as the need for standardized isolation techniques and the low abundance of certain RNA species, ongoing technological advancements may enable the integration of these biomarkers into clinical practice, ultimately enhancing early detection and personalized treatment plans.

Therapeutically, exosomes offer a promising vehicle for targeted drug delivery and cancer treatment in GI cancers. Their inherent ability to transfer functional molecules between cells can be harnessed to deliver therapeutic agents directly to tumor cells, minimizing off‐target effects and enhancing treatment efficacy. The engineering of exosomes to carry chemotherapeutic drugs, siRNAs, miRNA mimics, or inhibitors has shown success in inhibiting tumor growth, reducing metastasis, and overcoming drug resistance in preclinical models. Surface modifications of exosomes with targeting ligands, peptides, or aptamers have further improved their specificity and uptake by cancer cells. For example, exosomes modified with GE11 peptides or RGD peptides have demonstrated enhanced targeting and therapeutic effects in colorectal and pancreatic cancers, respectively. These advancements underscore the potential of exosome‐based therapies to provide a multifaceted approach to GI cancer treatment by combining direct tumor targeting with modulation of the tumor microenvironment.

Several exosome‐based diagnostic and therapeutic approaches for GI cancers are under investigation in clinical trials (**Table** **8**). After identifying lncRNA‐EV‐derived GC1 as a biomarker for early detection and monitoring of GC progression,^[^ ^30^ ^]^ the group at the Chinese People's Liberation Army (PLA) General Hospital extended their clinical trials to investigate its role in predicting immunotherapeutic outcomes (ClinicalTrials.gov identifier: NCT02536469). The expression levels of EV‐derived lncRNA‐GC1 were evaluated as a biomarker for predicting and monitoring immunotherapeutic outcomes in GC patients across four cohorts, including 84 patients in a retrospective training phase, 124 and 131 patients in retrospective validation cohorts, and 80 patients in a prospective validation phase.^[^ ^121^ ^]^ Patients with lower EV‐derived lncRNA‐GC1 levels demonstrated better PFS and OS, with median PFS ranging from 9.7 to 22.3 months and OS from 12.7 to 26.5 months, independent of PD‐L1 expression or CD8+ T cell infiltration. The biomarker showed stability under clinical conditions and achieved an AUC‐ROC of 0.7616 in predictive analyses, supporting its potential for guiding treatment decisions and improving GC immunotherapy outcomes.

**Table 8:** Overview of clinical trials registered on ClinicalTrials.gov investigating exosome‐based diagnostic and therapeutic systems for gastrointestinal (GI) cancer.

Identifier	Sponser	Disease	Purpose
NCT01779583	Hospital Miguel Servet	Gastric cancer (GC)	Characterize the molecular profile of GC‐derived exosomes and assess their potential as diagnostic, prognostic, and predictive biomarkers by evaluating their plasma levels and kinetics in advanced gastric cancer patients undergoing first‐line chemotherapy
NCT02393703	Memorial Sloan Kettering Cancer Center	Pancreatic cancer (PCa)	Analyze exosomes from blood and tissue samples of PCa patients and those with benign pancreatic disease to determine their role in disease recurrence and survival outcomes
NCT03032913	University Hospital, Bordeaux	PCa	Evaluate the diagnostic accuracy of onco‐exosome quantification in blood as a liquid biopsy approach for PCa by assessing their presence, correlation with clinical outcomes, and comparison with circulating tumor cells (CTCs)
NCT03250078	Nuvance Health	PCa	Develop a blood‐based screening test for PCa by isolating and analyzing circulating exosomes from individuals with a high genetic risk or family history of the disease
NCT03334708	Memorial Sloan Kettering Cancer Center	PCa	Develop a minimally invasive blood‐based test by analyzing exosomes and other biomarkers to enable early detection, surveillance, and treatment monitoring of PCa
NCT03432806	Memorial Sloan Kettering Cancer Center	Colorectal cancer (CRC)	Investigate exosome‐based biomarkers to improve the early detection of CRC and assess their role in predicting liver metastasis
NCT03874559	University of Kansas Medical Center	Rectal cancer (RC)	Characterize exosomal biomarker levels in patients with locally advanced rectal cancer undergoing neoadjuvant chemoradiation therapy and correlate these levels with pathologic response rates
NCT04394572	CHU de Reims	CRC	Identify new diagnostic protein markers for CRC by analyzing circulating tumor exosomes in patient blood samples
NCT04523389	Centre Hospitalier Universitaire Dijon	CRC	Investigate the prognostic role of circulating tumor‐derived exosomes and their contents as potential biomarkers for early prognosis in CRC patients
NCT05334849	Lin Chen	GC	Verify the function of circulating exosomal lncRNA‐GC1 in predicting and monitoring immunotherapy outcomes in patients with advanced gastric cancer
NCT05397548	Lin Chen	GC	Investigate the potential of circulating exosomal lncRNA‐GC1 as a biomarker for early detection and monitoring of gastric cancer
NCT05427227	Peking University	GI cancer	Use dynamic multiomics detection of plasma‐derived exosomes to explore the efficacy and mechanisms of anti‐HER2, immunotherapy, and anti‐CLDN18.2 treatments in advanced GI cancer patients
NCT05625529	Biological Dynamics	Pancreatic ductal adenocarcinoma (PDAC)	Evaluate the performance of the ExoVerita assay, which analyzes extracellular vesicle‐bound protein biomarkers, for early detection of PDAC in high‐risk or clinically‐suspicious populations
NCT06278064	Beijing Friendship Hospital	GC	Analyze plasma‐derived exosome proteins using advanced proteomics techniques to identify biomarkers for early diagnosis of upper GI cancers
NCT06342440	City of Hope Medical Center	CRC	Develop a highly sensitive and specific blood‐based assay for early detection of colorectal adenomas and cancer, utilizing cell‐free and exosome‐derived microRNA biomarkers
NCT06702891	Qilu Hospital of Shandong University	Gastric cardia cancer	Use integrative analysis of exosome‐mediated single‐cell transcriptomics and proteomics to explore heterogeneity, identify biomarkers, and discover potential therapeutic targets in gastric cardia cancer
NCT06777030	IRCCS Azienda Ospedaliero‐Universitaria di Bologna	PC	Characterize the content of extracellular vesicles (EVs)/exosomes in biological fluids of pancreatic cancer patients to identify potential new diagnostic and prognostic markers for the disease
NCT04483219	China Medical University, China	Metastatic colorectal adenocarcinoma	Evaluate the efficacy and safety of tyrosine kinase inhibitors combined with anti‐PD‐1 (programmed death 1) antibody in microsatellite stable/proficient mismatch repair metastatic CRC patients, with exosomes being analyzed as one of the exploratory biomarkers
NCT03608631	M.D. Anderson Cancer Center	PDAC	Determine the maximum tolerated dose and assess the safety of mesenchymal stromal cells‐derived exosomes loaded with KrasG12D siRNA (iExosomes) in treating patients with metastatic pancreatic cancer harboring the KrasG12D mutation
NCT06536712	Tehran University of Medical Sciences	RC	Evaluate the safety and efficacy of intraperitoneal administration of human placenta mesenchymal stem cell (MSC) derived exosomes in preventing early anastomotic leakage in patients undergoing low anterior resection for rectal cancer

While most of the clinical trials are focused on investigating the diagnostic capability of exosomes, exosome‐based therapeutics are also being applied in several clinical settings. As mentioned earlier, the group at M.D. Anderson Cancer Center has demonstrated that MSC‐derived exosomes loaded with siRNA targeting Kras^G12D^ reduced Kras^G12D^ mRNA levels, suppressed tumor growth, and significantly prolonged survival in murine models of PDAC.^[^ ^77^ ^]^ Following their initial findings, the group is now conducting a Phase 1 clinical trial to investigate the safety and efficacy of these exosomes in more detail, aiming to determine the maximum tolerated dose, assess dose‐limiting toxicities, and evaluate preliminary anti‐tumor activity of this therapeutic approach outcomes (ClinicalTrials.gov identifier: NCT03608631).

Despite these efforts, translating exosome‐based therapeutics into clinical applications presents several challenges. The scalability of exosome production remains a significant hurdle, as large quantities are required for therapeutic efficacy, and current isolation and purification methods are labor‐intensive and costly. Additionally, ensuring consistent loading of therapeutic cargoes and achieving targeted delivery without rapid clearance by the immune system are areas that require further research. Variability in exosome content, influenced by cellular origin, may impact therapeutic outcomes, necessitating standardized production protocols and thorough characterization. Addressing these challenges requires collaborative efforts to advance bioengineering techniques, expand knowledge of exosome biology, and develop scalable manufacturing and delivery solutions.

## 5 Conclusion

In summary, exosome‐based diagnostics and therapeutics have substantial potential to transform the management of GI cancers. By providing highly sensitive and specific biomarkers for early detection and enabling targeted, personalized treatment strategies, exosomes align closely with the principles of precision medicine. Integrating exosomal biomarkers into clinical workflows could significantly improve patient outcomes by facilitating earlier intervention and tailored therapies. Meanwhile, the therapeutic application of engineered exosomes presents a promising approach to overcoming current limitations in cancer treatment, such as drug resistance and off‐target toxicity. Continued investment in research and development, along with interdisciplinary collaboration, will be essential to addressing existing challenges and fully realizing the potential of exosome‐based approaches. As these efforts progress, exosomes may become integral to the future landscape of GI cancer diagnosis and therapy, ultimately improving survival rates and quality of life for patients.

## Conflict of Interest

The authors declare no conflict of interest.
